# Micronutrient status during pregnancy is associated with child immune status in rural Bangladesh

**DOI:** 10.1016/j.cdnut.2023.101969

**Published:** 2023-07-04

**Authors:** Da Kyung Jung, Sophia T. Tan, Caitlin Hemlock, Andrew N. Mertens, Christine P. Stewart, Md Ziaur Rahman, Shahjahan Ali, Rubhana Raqib, Jessica A. Grembi, Mohammed Rabiul Karim, Sunny Shahriar, Anjan Kumar Roy, Sarah Abdelrahman, Abul K. Shoab, Syeda L. Famida, Md Saheen Hossen, Palash Mutsuddi, Salma Akther, Mahbubur Rahman, Leanne Unicomb, Lisa Hester, Douglas A. Granger, Juergen Erhardt, Ruchira Tabassum Naved, Md Mahfuz Al Mamun, Kausar Parvin, John M. Colford, Lia C.H. Fernald, Stephen P. Luby, Firdaus S. Dhabhar, Audrie Lin

**Affiliations:** 1 Division of Epidemiology and Biostatistics, School of Public Health, University of California Berkeley, Berkeley, CA, United States; 2 Division of Infectious Diseases and Geographic Medicine, Stanford University, Stanford, CA, United States; 3 Institute for Global Nutrition, University of California Davis, Davis, CA, United States; 4 Environmental Interventions Unit, Infectious Diseases Division, icddr,b, Dhaka 1212, Bangladesh; 5 Department of Medicine, University of Maryland, Baltimore, MD USA; 6 Institute for Interdisciplinary Salivary Bioscience Research, University of California Irvine, Irvine, CA, United States; 7 VitMin Lab, Willstätt, Germany; 8 Health System and Population Studies Division, icddr,b, Dhaka, Bangladesh; 9 Division of Community Health Sciences, School of Public Health, University of California, Berkeley, Berkeley, CA, United States; 10 Department of Psychiatry & Behavioral Sciences, Department of Microbiology and Immunology, Sylvester Comprehensive Cancer Center, Miller School of Medicine, University of Miami, Miami, FL, United States; 11 Department of Microbiology and Environmental Toxicology, University of California, Santa Cruz, CA, United States

**Keywords:** pregnancy, maternal micronutrients, child immune status, vitamin A, iron

## Abstract

**Background:**

Poor immune function increases children’s risk of infection and mortality. Several maternal factors during pregnancy may affect infant immune function during the postnatal period.

**Objectives:**

We aimed to evaluate whether maternal micronutrients, stress, estriol, and immune status during the first or second trimester of pregnancy were associated with child immune status in the first two years after birth.

**Methods:**

We conducted observational analyses within the water, sanitation, and hygiene (WASH) Benefits Bangladesh randomized controlled trial. We measured biomarkers in 575 pregnant women and postnatally in their children. Maternal biomarkers measured during the first and second trimester of pregnancy included nutrition status via vitamin D (25-hydroxy-D [25(OH)D]), ferritin, soluble transferrin receptor (sTfR), and retinol-binding protein (RBP); cortisol; estriol. Immune markers were assessed in pregnant women at enrollment and their children at ages 14 and 28 mo, including C-reactive protein (CRP), alpha-1-acid glycoprotein (AGP), and 13 cytokines (including IFN-γ). We generated a standardized sum score of log-transformed cytokines. We analyzed IFN-γ individually because it is a critical immunoregulatory cytokine. All outcomes were prespecified. We used generalized additive models and reported the mean difference and 95% confidence intervals at the 25th and 75th percentiles of exposure distribution.

**Results:**

At child age 14 mo, concentrations of maternal RBP were inversely associated with the cytokine sum score in children (-0.34 adjusted difference between the 25th and 75th percentile [95% confidence interval -0.61, -0.07]), and maternal vitamin A deficiency was positively associated with the cytokine sum score in children (1.02 [0.13, 1.91]). At child age of 28 mo, maternal RBP was positively associated with IFN-γ in children (0.07 [0.01, 0.14]), whereas maternal vitamin A deficiency was negatively associated with child AGP (-0.07 [-0.13, -0.02]). Maternal iron deficiency was associated with higher AGP concentrations in children at age 14 mo (0.13 [0.04, 0.23]), and maternal sTfR concentrations were positively associated with child CRP concentrations at age 28 mo (0.18 [0, 0.36]).

**Conclusion:**

Maternal deficiencies in vitamin A or iron during the first 2 trimesters of pregnancy may shape the trajectory of a child’s immune status.

## Introduction

The immune system protects children from infection and mortality. Optimal immune development includes maturation of the innate and adaptive immune systems ante- and postnatally [1]. One important innate mechanism of defense against pathogens is the inflammatory response. Although a short-term inflammatory response allows the body to defend itself, sustained inflammation can be costly. Chronic and abnormally high inflammation in infants can lead to poor growth, chronic diseases, and impaired neurodevelopment [2,3]. If continued into adulthood, low-grade inflammation may contribute to the development of chronic disease [1,4].

It is important to assess the impact of antenatal factors on child immune health because adult health outcomes are often rooted upstream through *in utero* exposures that have long-term effects [5] and can be modified by targeted interventions. For example, fetal exposure to a variety of factors, including pollution, toxins, poverty, inflammation, and stress during pregnancy, can all contribute to inflammation in the child during the postnatal period [2].

A healthy maternal immune system during pregnancy yields robust responses to stressors like pathogens and appropriate regulation of such responses. Maternal immune health is vital in the development of the child’s immune system due to direct and indirect interactions between their immune systems. For example, bidirectional transfer of IL-6 occurs between the mother and fetus [6]. A study in Zimbabwe found that inflammation levels, measured by alpha-1-acid glycoprotein (AGP) and CRP, in women and their children were associated throughout infancy; however, this finding could also be due to exposure to common environmental stressors [7]. Furthermore, prenatal pathogen infection in women can cause immune dysregulation in their children that can persist into adulthood [8].

Maternal micronutrients during pregnancy are vital for the immune system of the child. Studies link maternal vitamin D status to lower risk of infections and immune-mediated diseases during infancy [9]. Maternal vitamin A is important for ocular, skeletal, and immune health [[10], [11], [12], [13]]. Vitamin A appears to have a dose-differential effect on the inhibition and upregulation of immune cell synthesis and differentiation [[14], [15], [16]]. Trials in rural Nepal found that maternal vitamin A supplementation in a vitamin A-deficient population was positively associated with adaptive immune function in their preadolescent children [17]. We are unaware of any studies assessing maternal iron status during the first trimester of pregnancy—the period of fetal organogenesis—and immune biomarkers during childhood. In one longitudinal study in the United Kingdom, maternal iron status during pregnancy was associated with indirect measures of child immune status, such as wheezing and atopic sensitization [18]. Decreased maternal ferritin, which indicates the amount of iron stored during the first trimester, was associated with lower lung function measurements in children. Higher maternal sTfR, which reflects higher demand for iron in cells at delivery, was associated with higher risk of atopic sensitization in children. Thus, insufficient maternal iron during pregnancy may be associated with the upregulation of child immune markers.

The maternal endocrine system during pregnancy also contributes to child health and immune function [19]. Estriol, the main estrogen produced during pregnancy, is positively associated with child birth weight [20]. Cortisol, a stress hormone produced by the adrenal glands, is released when the hypothalamic pituitary adrenal (HPA) axis is activated. Throughout a normal course of pregnancy, cortisol concentrations increase 2 to 4 times their prepregnancy amounts due to physiologic responses to hormonal changes. However, excessive concentrations of cortisol may be detrimental to fetal immune system development because transplacental exposure to maternal cortisol may chronically activate the offspring’s HPA axis [21]. This, in turn, leads to hyperactivity of proinflammatory cytokine production and blunted responses to regulatory mechanisms. To our knowledge, no studies have examined the association between stress during pregnancy and immune function in the child.

This study, conducted in rural Bangladesh, where there are high levels of pediatric stunting and infection [22], evaluated whether maternal nutrition, hormones, and immune status measured during the first or second trimester of pregnancy were associated with offspring's immune status in early childhood.

## Methods

### Study site and design

We performed observational analyses nested within the water, sanitation, and hygiene (WASH) Benefits Bangladesh study, a cluster-randomized controlled trial (RCT) in rural villages of the Gazipur, Kishoreganj, Mymensingh, and Tangail districts of Bangladesh from May 2012 to March 2016 [23]. Trial methods are detailed elsewhere [22]. The trial included 7 arms: control, water (chlorinated drinking water) only, sanitation (upgraded sanitation) only, handwashing (promoting handwashing with soap) only, combined water + sanitation + handwashing (WSH), nutrition (child nutrition counseling and lipid-based nutrient supplements) only, and nutrition + WSH (N+WSH) [23]. For our analyses, mother-child dyads were selected from the control and N+WSH arms of the environmental enteric dysfunction substudy, which collected additional biological samples [24].

### Participants

Households in rural Bangladesh are often organized into compounds around community resources. A child born to an enrolled pregnant woman was eligible (index child) if the mother planned to reside in the study village for the next 2 y. From each compound, only 1 pregnant woman was enrolled, but both children were enrolled if she gave birth to twins [23].

Power calculations were performed and can be found in the Supplementary Materials.

### Data collection and procedures

Maternal characteristics, such as age and education level, were measured in women at enrollment during either their first or second trimester. Child measurements at ages 14 and 28 mo included anthropometric measurements such as length-for-age (LAZ), weight-for-age (WAZ), weight-for-length (WLZ), and head-circumference-for-age (HCZ) Z-scores. Household measurements included the Household Food Insecurity Access Scale (HFIAS) at enrollment.

Exposure measurements were taken in the first or second trimester before mothers were randomly assigned to an arm of the WASH Benefits trial. Between May 2012 and July 2013, research assistants used trace metal-free certified needles and tubes (Sarstedt) to collect 10 mL blood samples from the women. Blood samples were stored at -80ºC. We evaluated maternal nutrition status through serum vitamin D (25-hydroxy-D [25(OH)D]), ferritin, sTfR, and RBP; maternal hormones through serum cortisol and estriol; and maternal immune status through serum CRP, serum alpha-1-acid glycoprotein (AGP), and 13 plasma cytokine outcomes [interleukin-1β (IL-1β), IL-6, tumor necrosis factor-α (TNF-α), IL-2, IL-12p70 (IL-12), interferon-γ (IFN-γ), IL-4, IL-5, IL-13, IL-17A, IL-21, IL-10, granulocyte-macrophage colony-stimulating factor (GM-CSF)].

Outcome measurements were assessed in children at ages 14 and 28 mo via venous blood samples collected in the manner described above. Immune status was assessed by identical CRP, AGP (age 14 mo only), and cytokine measures as were used for the mothers.

### Laboratory methods

Maternal vitamin D was measured via the Roche Kit by electrochemiluminescence binding assay using the Roche automated immunoanalyzer (cobas e601) at the Nutritional Biochemistry Lab of the International Centre for Diarrhoeal Disease Research, Bangladesh (icddr,b). Ferritin, sTfR, and RBP were assessed via the sandwich ELISA assay at the VitMin Lab (Willstätt, Germany) [25]. Micronutrients were evaluated as continuous exposures and as binary exposures: vitamin A deficiency (RBP <0.7 μmol/L), vitamin D deficiency (25-OH-VitD < 30 nmol/L), and iron deficiency (ferritin <12 μg/L or sTfR >8.3 mg/L) [26]. Because ferritin, sTfR, and RBP are affected by the acute-phase response, we corrected these values for inflammation (CRP and AGP) using the BRINDA method [27], as published in the primary micronutrients analysis in the WASH Benefits study [26].

Maternal serum cortisol concentrations were measured via the DetectX® Cortisol Immunoassay kit, following kit instructions. Maternal serum estriol was measured via enzyme immunoassay (IBL-America Free Estriol ELISA). Both hormone levels were measured at icddr,b.

AGP and CRP measurements at child age 14 mo were performed at icddr,b. ELISA kit protocols were followed with initial dilutions of 1:100 for CRP and 1:10000 for AGP (R&D Systems, Minneapolis, MN). All out-of-range specimens were run again at higher or lower dilutions. The coefficient of variation for the above assays was <10%.

Maternal AGP and CRP, as well as child CRP (age 28 mo) measurements, were completed at the VitMin Lab (Willstätt, Germany) [25]*.*

The 13 plasma cytokine outcomes were measured at the University of Maryland via multiplex Luminex technology (Millipore kit HSTCMAG-28SK). Samples were assayed in duplicate, and each plate contained high and low controls. 200 μL of assay buffer was used to wet a 96-well plate (Greiner). The plate was placed on a shaker for 10 min and then decanted. The following was added to each well: 25 μL of assay buffer and 25 μL of sample, controls, or standards. 25 μL of a mixture containing cytokines (1:50 dilution) was conjugated to beads. Next, the beads were added to the plate, which was placed on a shaker at 4°C overnight. The next day, the following sequence of steps was completed 3 times: the plate was placed on a magnetic washer, 200 μL of wash buffer was added to each well, and the plate was placed on a shaker at 500 rpm for 1 min. After the last decanting step, 25 μL of detection antibody was added, and the plate was placed on a shaker for 1 h at room temperature. Then, 25 μL of Phycoerythrin (1:25 dilution) was added to each well, and the plate was placed on the shaker for 30 min. The plate was washed 3 times, and 150 μL of Sheath Fluid was added to each well. Finally, a Luminex 100 reader read the plate. Data calculations were performed with Bio-Rad Bio-Plex Software. The coefficient of variation for the cytokine assay was <20%. Distributions of immune markers were skewed, so all cytokine, CRP, and AGP values were log-transformed.

### Construction of immune sum score

The 13 log-transformed cytokines values were scaled to generate Z-scores of each marker. Because these cytokine Z-scores showed positive pairwise correlations (Supplementary Figure 1), the Z-scores were added to create a sum score of the 13 cytokines to represent total inflammation [4]. Additionally, we used k-nearest neighbor imputation for all missing immune marker values to reduce missing-data bias.

We additionally prespecified and analyzed IFN-γ as an individual cytokine because it is a critical regulatory cytokine.

### Statistical analyses

Analyses were prespecified, registered on Open Science Framework, and conducted in R (version 4.1.1) (https://osf.io/g2u8f/). Data and scripts are publicly available on Open Science Framework.

First, we conducted exploratory data analyses that plotted the association between each maternal biomarker at enrollment and child immune biomarkers at ages 14 and 28 mo. Due to the exploratory nature of these analyses, both the strength of relationships between individual biomarkers (*P* < 0.05) and the consistency in the direction of individual biomarkers within each related biomarker group were considered during interpretation. In addition to utilizing typical corrections for false discovery rate (FDR) to estimate the probability of an individual result being due to chance, we also aimed to determine whether multiple measures of a related exposure-outcome domain (e.g., maternal iron concentrations and child immune status) reflected an underlying relationship. Therefore, if relationships within one domain of exposure-outcome clustered closely above and below the null hypothesis, we concluded that an individual measure with statistical significance in that cluster may have been due to spurious association from repeated testing. We used the Benjamini-Hochberg procedure to adjust for multiple testing by controlling the FDR within each maternal pregnancy exposure (FDR <0.2).

Because the relationship between the maternal biomarkers and child immune status could be nonlinear, we summarized mean child immune status at ages 14 and 28 mo across the distributions of maternal nutrition, stress, immune status, and estriol using natural smoothing splines. These generalized additive models were both unadjusted and adjusted for confounders, including child age, sex, and prescreened covariates found to be significantly related to the outcome in bivariate analyses. The prescreening process used the likelihood ratio test, and covariates were included in the analysis if significantly related to each outcome (*P* < 0.20), whereas they were excluded if the covariate had little variation in the study population (prevalence <5%). Additionally, the cortisol results were adjusted using the time that the sample was placed on a cold chain as a proxy for time of day to control for the cortisol awakening response. Full lists of covariates are included in table footnotes.

We plotted the generalized additive model curves between exposures and outcomes and their 95% simultaneous confidence intervals. Also, we estimated the differences and confidence intervals for the 25th and 75th percentiles of each exposure distribution.

### *Post-hoc* analyses

To elucidate the relationship between maternal vitamin A and iron measured during pregnancy with childhood immune status, we created log-transformed ratios of child cytokine values as post-hoc outcomes: intracellular pathogen defense Th1 (IL-12 + IFN-γ) / extracellular pathogen defense Th2 (IL-4 + IL-5 + IL-13), proinflammatory (IL-1β + IL-6 + TNF-α) / immunoregulatory (IL-10), Th1 / IL-10, and Th2 / IL-10 at both 14 and 28 mo. The purpose of these ratios was to assess the overall proinflammatory versus immunoregulatory environment. Because the overall cytokine sum score includes all cytokines, our prespecified measure of the sum score may have obscured the shifts in proinflammatory and immunoregulatory responses by averaging them.

In the N+WSH treatment arm, mothers and children received nutrition recommendations, and children between the ages of 6 to 24 mo received micronutrient-fortified lipid-based nutrient supplementation [24]. Such nutritional interventions could have differentially affected the relationship between maternal micronutrients and child immune status between those who received the intervention compared to those who did not. Therefore, effect measure modification was tested by the intervention arm (N+WSH and control) for the maternal micronutrients results.

We assessed acute respiratory illness (defined as caregiver-reported persistent cough, panting, wheezing, or difficulty breathing in the past 7 d) and diarrhea (defined as caregiver-reported 3 or more loose stools within a 24-h period in the past 7 d) in children at 14 mo and 28 mo. Since recent illness can impact the levels of ongoing immune system activation, we conducted an analysis where we included only children without recent caregiver-reported diarrhea or acute respiratory infection.

Lastly, we added low maternal vitamin A status (RBP <1.05 μmol/L) during pregnancy as an exposure.

### Ethics

All expectant women and the primary caregivers of all children provided written informed consent before enrollment. The study protocols were approved by human subjects committees at the International Centre for Diarrhoeal Disease Research, Bangladesh (icddr,b), the University of California, Berkeley, and Stanford University. The parent trial was overseen by a data safety monitoring committee convened by icddr,b [22] and was registered at ClinicalTrials.gov (NCT01590095).

## Results

### Enrollment characteristics

**FIGURE 1:**
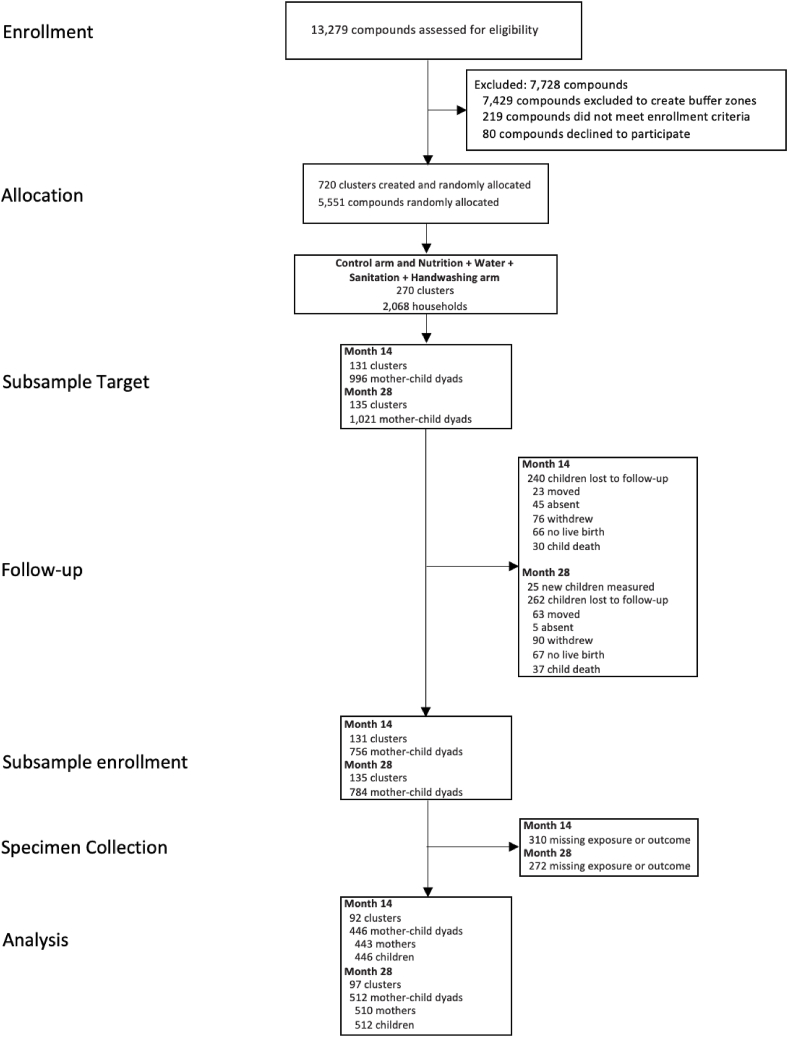
Flowchart of participants’ progress through enrollment, sample collection, and statistical analysis.

The parent trial enrolled 5551 pregnant women and their offspring. The substudy enrolled 756 children at age 14 mo and 784 children at age 28 mo; immune marker measurements were available from 446 (59%) children at 14 mo and 512 (65%) at 28 mo (Figure 1). This analysis includes 578 mother-child dyads (575 mothers and 578 children) with both pregnancy data and child immune marker measurements at either 14 mo or 28 mo. Three hundred eighty dyads had child immune marker measurements at both 14 and 28 mo.

At sample collection, the median age of the pregnant women was age 24 y (IQR 20, 27; Table 1). At the time of maternal measurement, the median gestational age of the children was 21.9 wk (IQR 17.3, 25.9). The women’s education levels were measured by years of schooling, for which the median was 6 y (IQR 4, 9). We found that 162 households (28%) were reported as food-insecure on the HFIAS.

**TABLE 1:** Enrollment characteristics.

			*n* (%) or median (IQR)
Child		Female	293 (51%)
	Anthropometry (3 mo)	Length-for-age *Z* score	-1.29 (-2.04, -0.48)
		Weight-for-age *Z* score	-1.21 (-1.84, -0.51)
		Weight-for-length *Z* score	-0.26 (-1.19, 0.5)
		Head circumference-for-age Z score	-1.83 (-2.49, -1.13)
	Anthropometry (14 mo)	Length-for-age *Z* score	-1.44 (-2.22, -0.82)
		Weight-for-age *Z* score	-1.39 (-2.07, -0.8)
		Weight-for-length *Z* score	-1 (-1.68, -0.32)
		Head circumference-for-age *Z* score	-1.81 (-2.43, -1.23)
	Anthropometry (28 mo)	Length-for-age *Z* score	-1.6 (-2.35, -1.01)
		Weight-for-age *Z* score	-1.57 (-2.13, -0.96)
		Weight-for-length *Z* score	-0.98 (-1.6, -0.36)
		Head circumference-for-age *Z* score	-1.79 (-2.37, -1.25)
	Diarrhea (14 mo)	Caregiver-reported 7-d recall	82 (16%)
	Diarrhea (28 mo)	Caregiver-reported 7-d recall	42 (8%)
	Acute respiratory illness (14 mo)	Caregiver-reported 7-d recall	154 (31%)
	Acute respiratory illness (28 mo)	Caregiver-reported 7-d recall	131 (25%)
Mother		Age (y)	24 (20, 27)
		Gestational age (wk)	21.86 (17.29, 25.86)
	Anthropometry at enrollment	Height (cm)	149.93 (146.67, 153.71)
	Education	Schooling completed (y)	6 (4, 9)
	Depression (14 mo)	CESD-20^1^ score	10 (6, 16)
	Depression (28 mo)	CESD-20^1^ score	9 (5, 17)
	Perceived stress (28 mo)	Perceived Stress Scale score	14 (11, 18)
	Intimate partner violence	Any lifetime exposure	288 (57%)
Household	Household Food Insecurity	Food-insecure households	162 (28%)

1 CESD-20 = Center for Epidemiologic Studies Depression Scale Revised

At age 14 mo, median LAZ was −1.44, median WAZ was −1.39, median WLZ was −1.00, and median HCZ was −1.81 (Table 1). At 28 mo, Z-scores of anthropometric measurements remained similar. 16% of children and 8% of children reported having diarrhea in the past 7 d at 14 mo and 28 mo, respectively. 31% of children reported having acute respiratory illness in the past 7 d at age 14 mo. 25% of children reported having acute respiratory illness at age 28 mo.

### Maternal exposure levels

During pregnancy, 108 women (19%) were vitamin D deficient, and 12 women (2%) were vitamin A deficient (Supplementary Table 1). In terms of iron status, the medians for inflammation-adjusted ferritin and sTfR concentrations were 24.64 μg/L (IQR 12.89, 48.33) and 4.28 mg/L (IQR 3.62, 5.42), respectively. One hundred thirty-four women (23%) were iron deficient. Among those women, 106 (79.1%) were considered deficient because of their ferritin concentrations only, 7 (5.2%) due to their sTfR concentrations only, and 21 (15.7%) were based on both parameters. Therefore, most iron deficiency in mothers was ascertained by low ferritin rather than high sTfR. For maternal hormones, the median of cortisol was 18.9 μg/dL (IQR 12.36, 26.16), and the median of estriol was 3.87 ng/mL (2.05, 5.59). Maternal measurements of key cytokines in pg/mL include IL-6 (median 2.29, IQR (1.26, 3.55), IL-10 median 6.77, IQR 3.92, 11.21), and IFN-γ [median 7.27, IQR (5.22, 10.04)]. The median maternal cytokine sum score was 0.12 (IQR -0.64, 0.65). The median AGP concentration was 0.44 g/L (IQR 0.33, 0.57), and the median CRP concentration was 1.94 mg/L (IQR 0.91, 4.06).

### Child outcome levels

The median child cytokine sum scores were 0.04 (IQR -0.69, 0.63) at 14 mo and 0.01 at 28 mo (IQR -0.66, 0.62; Supplementary Table 2). AGP was measured at 14 mo only, and the median was 1.02 g/L (IQR 0.77, 1.4). CRP was measured as 1.17 mg/L (IQR 0.39, 3.39) at 14 mo and 0.01 mg/L (IQR -0.66, 0.62) at 28 mo.

### Maternal micronutrients and child immune status

**FIGURE 2:**
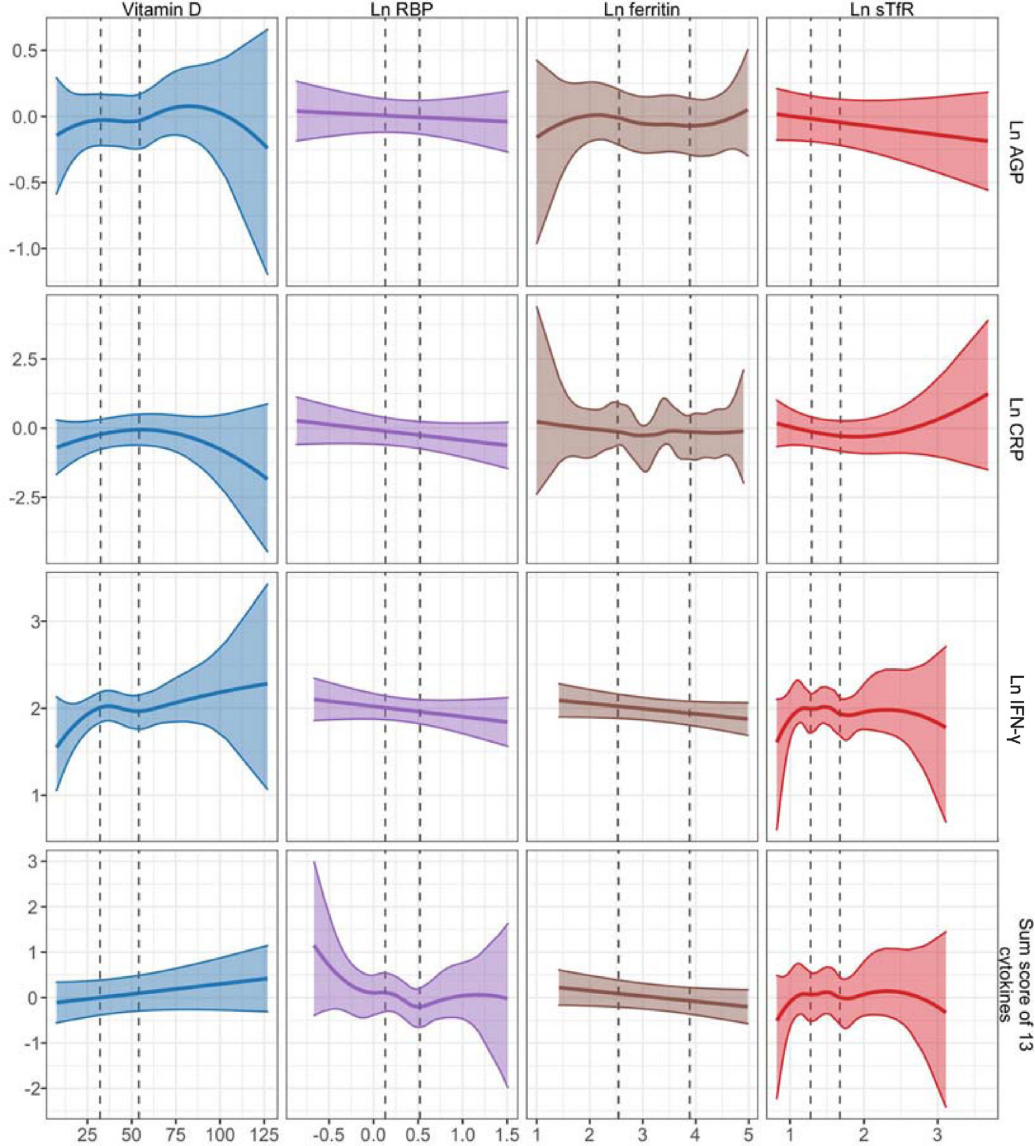
Spline plots for maternal micronutrient status and child immune status at 14 mo.

**FIGURE 3:**
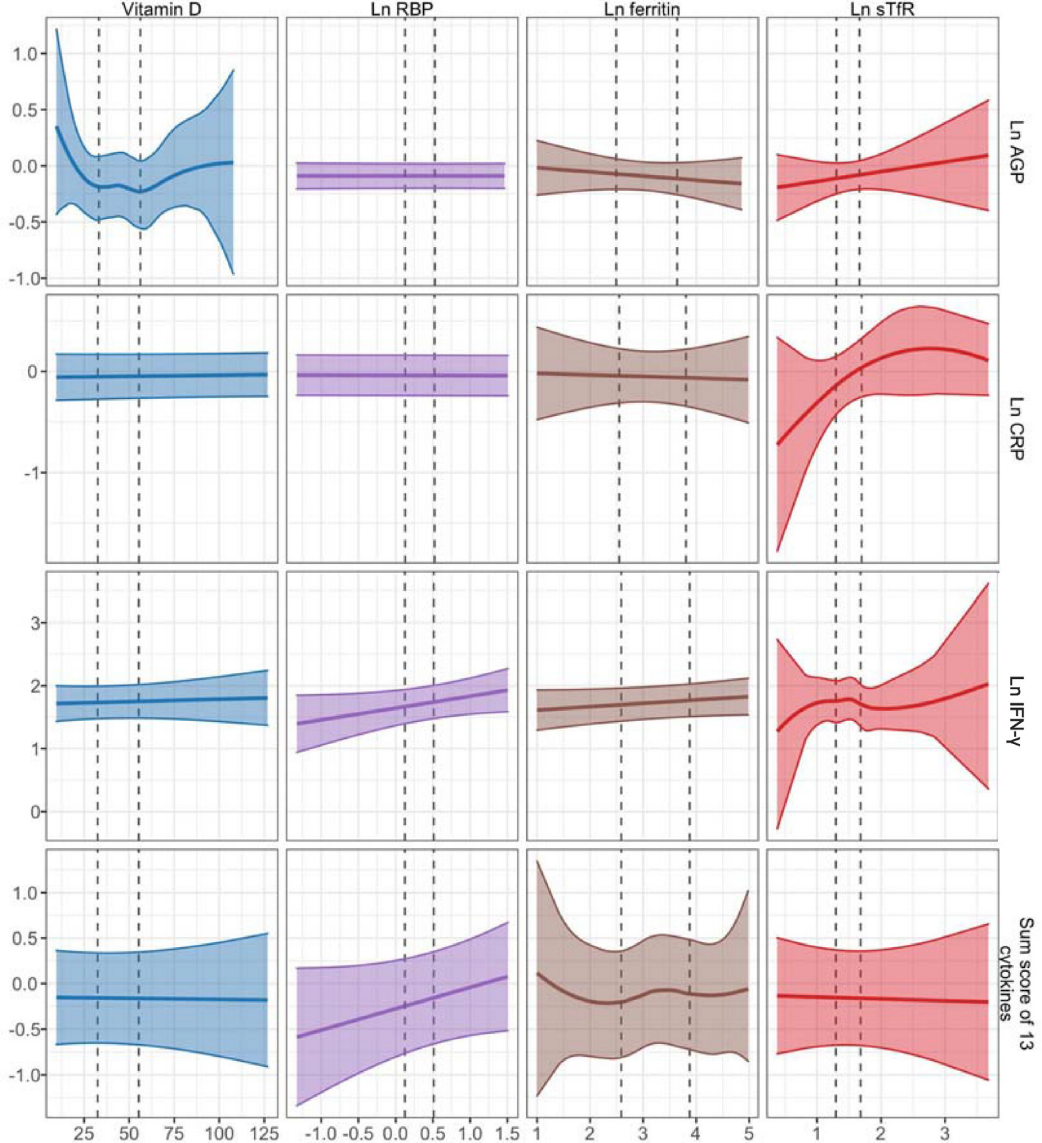
Spline plots for maternal micronutrient status and child immune status at 28 mo.

**FIGURE 4:**
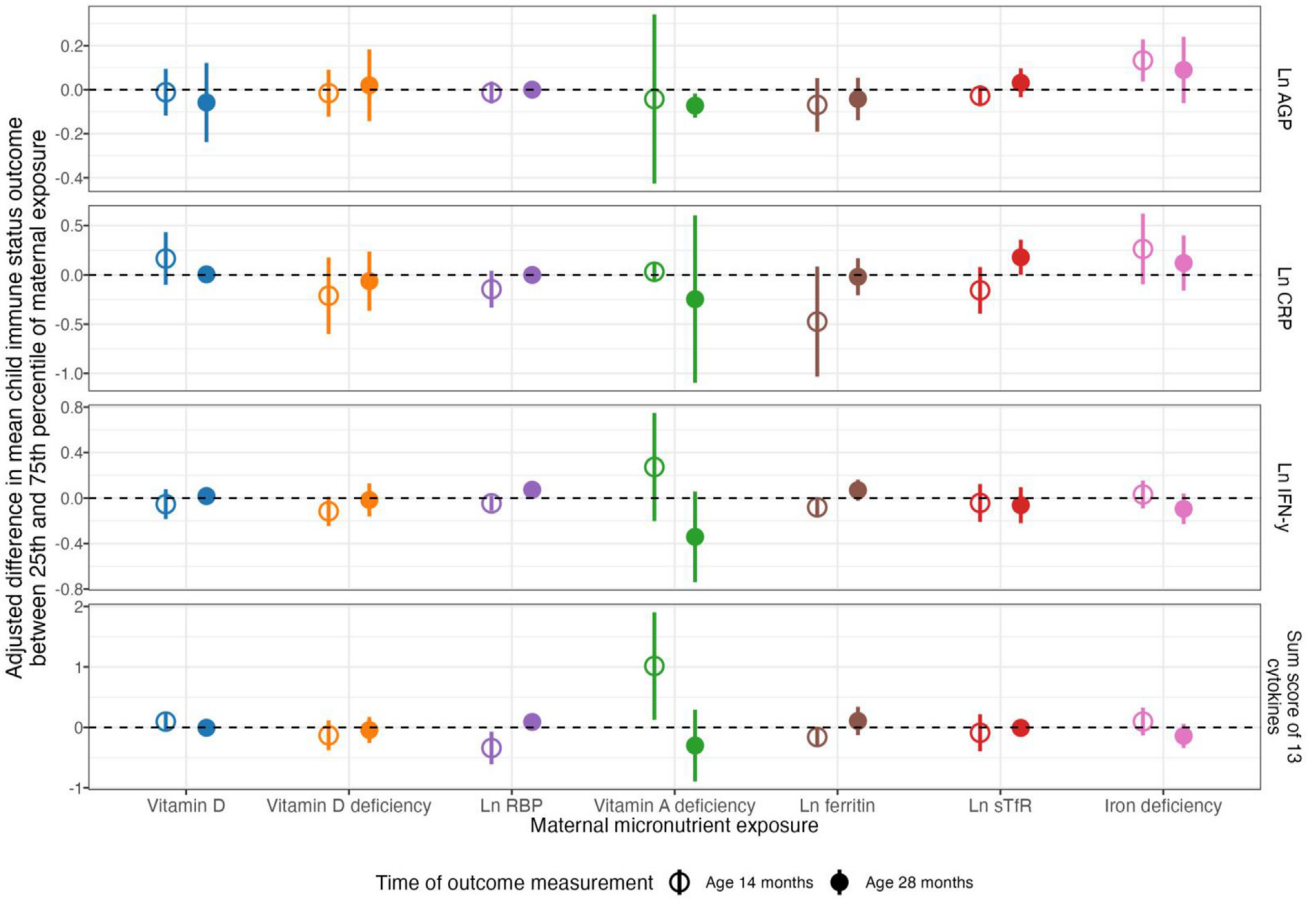
A visual summary of the associations between maternal micronutrient status and child immune status.

Concentrations of maternal RBP during the first or second trimester of pregnancy were inversely associated with cytokine sum scores in children at 14 mo (-0.34 adjusted difference between the 25th and 75th percentile [95% CI -0.61, -0.07]; Table 2, Supplementary Table 3). Maternal RBP was positively associated with IFN-γ at 28 mo (0.07 log pg/mL adjusted difference [95% CI 0.01, 0.14]). Overall, the association between maternal RBP and child immune status was negative at 14 mo and positive at 28 mo. In coherence with the RBP results, maternal vitamin A deficiency was positively associated with child cytokine sum scores at 14 mo (1.02 adjusted difference [0.13, 1.91]; Table 2, Supplementary Table 3). Maternal vitamin A deficiency was negatively associated with AGP concentration at 28 mo (-0.07 log g/L adjusted difference [-0.13, -0.02]). Maternal ferritin concentrations were not significantly associated with child immune markers, but maternal sTfR concentrations were positively associated with CRP concentrations in children at 28 mo with *P* value of < 0.05 (0.18 log mg/L adjusted difference [0, 0.36]). Maternal iron deficiency was associated with elevated child AGP concentrations at 14 mo (0.13 log g/L adjusted difference [0.04, 0.23]) (Table 2, Supplementary Table 3, FIGURE 2, FIGURE 3). We observed no significant associations between maternal vitamin D and child immune status at 14 and 28 mo (Figure 4). No associations were significant after FDR correction.

**TABLE 2:** Maternal micronutrients and child immune status.

Maternal micronutrients and child immune status	Outcome	*n*	25th percentile	75th percentile	Outcome, 75th percentile vs. 25th percentile			
					Adjusted			
					Predicted outcome at 25th percentile	Predicted outcome at 75th percentile	Coefficient (95% CI)	*P*
Vitamin D (nmol/L)	Ln AGP Age 14 mo (g/L)	421	32.53	54.54	-0.03	-0.04	-0.01 (-0.12, 0.09)	0.84
	Ln CRP Age 14 mo (mg/L)	367	32.48	54.22	-0.23	-0.06	0.17 (-0.1, 0.43)	0.23
	Ln IFN-γ Age 14 mo (pg/mL)	408	32.12	54.06	2.01	1.96	-0.05 (-0.18, 0.08)	0.43
	Sum score of 13 cytokines Age 14 mo	407	32.12	54.08	0	0.1	0.1 (-0.04, 0.23)	0.15
	Ln AGP Age 28 mo (g/L)	250	33.18	56.25	-0.2	-0.26	-0.06 (-0.24, 0.12)	0.54
	Ln CRP Age 28 mo (mg/L)	496	32.57	55.49	-0.05	-0.05	0.01 (-0.01, 0.02)	0.61
	Ln IFN-γ Age 28 mo (pg/mL)	487	32.48	55.36	1.73	1.75	0.02 (-0.06, 0.1)	0.67
	Sum score of 13 cytokines Age 28 mo	487	32.48	55.36	-0.16	-0.16	-0.01 (-0.12, 0.11)	0.93
Vitamin D deficiency	Ln AGP Age 14 mo (g/L)	421	0	1	-0.02	-0.04	-0.02 (-0.12, 0.09)	0.78
	Ln CRP Age 14 mo (mg/L)	367	0	1	-0.13	-0.34	-0.21 (-0.6, 0.18)	0.29
	Ln IFN-γ Age 14 mo (pg/mL)	408	0	1	2.02	1.9	-0.12 (-0.25, 0.01)	0.08
	Sum score of 13 cytokines Age 14 mo	407	0	1	0.08	-0.05	-0.13 (-0.38, 0.12)	0.3
	Ln AGP Age 28 mo (g/L)	250	0	1	-0.1	-0.08	0.02 (-0.14, 0.18)	0.82
	Ln CRP Age 28 mo (mg/L)	496	0	1	-0.04	-0.1	-0.06 (-0.36, 0.24)	0.69
	Ln IFN-γ Age 28 mo (pg/mL)	487	0	1	1.74	1.72	-0.02 (-0.16, 0.13)	0.83
	Sum score of 13 cytokines Age 28 mo	487	0	1	-0.14	-0.18	-0.04 (-0.26, 0.17)	0.72
Ln RBP (μmol/L)	Ln AGP Age 14 mo (g/L)	421	0.13	0.52	0.01	-0.01	-0.01 (-0.06, 0.04)	0.63
	Ln CRP Age 14 mo (mg/L)	367	0.13	0.52	-0.11	-0.25	-0.15 (-0.33, 0.04)	0.13
	Ln IFN-γ Age 14 mo (pg/mL)	408	0.13	0.52	2.01	1.96	-0.05 (-0.11, 0.02)	0.17
	Sum score of 13 cytokines Age 14 mo	407	0.13	0.52	0.12	-0.22	-0.34 (-0.61, -0.07)	0.01
	Ln AGP Age 28 mo (g/L)	250	0.13	0.53	-0.09	-0.09	0 (-0.01, 0.01)	1
	Ln CRP Age 28 mo (mg/L)	496	0.13	0.52	-0.04	-0.04	0 (0, 0)	0.41
	Ln IFN-γ Age 28 mo (pg/mL)	487	0.12	0.51	1.67	1.74	0.07 (0.01, 0.14)	0.03
	Sum score of 13 cytokines Age 28 mo	487	0.12	0.51	-0.25	-0.16	0.09 (-0.01, 0.19)	0.07
Vitamin A deficiency	Ln AGP Age 14 mo (g/L)	421	0	1	-0.03	-0.07	-0.04 (-0.43, 0.34)	0.84
	Ln CRP Age 14 mo (mg/L)	367	0	1	-0.13	-0.1	0.03 (-0.07, 0.13)	0.54
	Ln IFN-γ Age 14 mo (pg/mL)	408	0	1	1.98	2.25	0.27 (-0.2, 0.75)	0.26
	Sum score of 13 cytokines Age 14 mo	407	0	1	-0.01	1.01	1.02 (0.13, 1.91)	0.02
	Ln AGP Age 28 mo (g/L)	250	0	1	-0.09	-0.16	-0.07 (-0.13, -0.02)	0.01
	Ln CRP Age 28 mo (mg/L)	496	0	1	-0.05	-0.3	-0.25 (-1.1, 0.6)	0.58
	Ln IFN-γ Age 28 mo (pg/mL)	487	0	1	1.73	1.39	-0.34 (-0.74, 0.06)	0.09
	Sum score of 13 cytokines Age 28 mo	487	0	1	-0.16	-0.46	-0.3 (-0.89, 0.3)	0.33
Ln ferritin (μg/L)	Ln AGP Age 14 mo (g/L)	421	2.55	3.89	-0.01	-0.08	-0.07 (-0.19, 0.05)	0.27
	Ln CRP Age 14 mo (mg/L)	367	2.53	3.9	0.15	-0.33	-0.47 (-1.03, 0.08)	0.1
	Ln IFN-γ Age 14 mo (pg/mL)	408	2.53	3.88	2.02	1.94	-0.08 (-0.17, 0.01)	0.07
	Sum score of 13 cytokines Age 14 mo	407	2.54	3.88	0.09	-0.07	-0.16 (-0.32, 0)	0.06
	Ln AGP Age 28 mo (g/L)	250	2.49	3.64	-0.07	-0.12	-0.04 (-0.14, 0.05)	0.4
	Ln CRP Age 28 mo (mg/L)	496	2.55	3.81	-0.04	-0.06	-0.02 (-0.21, 0.17)	0.85
	Ln IFN-γ Age 28 mo (pg/mL)	487	2.59	3.88	1.7	1.77	0.07 (-0.02, 0.16)	0.14
	Sum score of 13 cytokines Age 28 mo	487	2.59	3.88	-0.23	-0.12	0.11 (-0.13, 0.34)	0.38
Ln sTfR (mg/L)	Ln AGP Age 14 mo (g/L)	421	1.28	1.67	-0.02	-0.04	-0.03 (-0.08, 0.02)	0.26
	Ln CRP Age 14 mo (mg/L)	367	1.29	1.68	-0.13	-0.29	-0.16 (-0.39, 0.08)	0.19
	Ln IFN-γ Age 14 mo (pg/mL)	408	1.28	1.67	1.95	1.9	-0.04 (-0.21, 0.12)	0.63
	Sum score of 13 cytokines Age 14 mo	407	1.28	1.67	0.02	-0.07	-0.09 (-0.4, 0.22)	0.58
	Ln AGP Age 28 mo (g/L)	250	1.3	1.66	-0.11	-0.08	0.03 (-0.03, 0.1)	0.35
	Ln CRP Age 28 mo (mg/L)	496	1.3	1.7	-0.14	0.04	0.18 (0, 0.36)	0.05
	Ln IFN-γ Age 28 mo (pg/mL)	487	1.29	1.68	1.75	1.69	-0.06 (-0.22, 0.1)	0.45
	Sum score of 13 cytokines Age 28 mo	487	1.29	1.68	-0.15	-0.16	-0.01 (-0.11, 0.09)	0.89
Iron deficiency	Ln AGP Age 14 mo (g/L)	421	0	1	-0.03	0.1	0.13 (0.04, 0.23)	0.01
	Ln CRP Age 14 mo (mg/L)	367	0	1	-0.21	0.05	0.26 (-0.1, 0.62)	0.15
	Ln IFN-γ Age 14 mo (pg/mL)	408	0	1	1.98	2.01	0.03 (-0.09, 0.15)	0.63
	Sum score of 13 cytokines Age 14 mo	407	0	1	-0.02	0.08	0.1 (-0.13, 0.33)	0.42
	Ln AGP Age 28 mo (g/L)	250	0	1	-0.13	-0.04	0.09 (-0.06, 0.24)	0.25
	Ln CRP Age 28 mo (mg/L)	496	0	1	-0.09	0.03	0.12 (-0.16, 0.4)	0.41
	Ln IFN-γ Age 28 mo (pg/mL)	487	0	1	1.76	1.67	-0.09 (-0.23, 0.04)	0.17
	Sum score of 13 cytokines Age 28 mo	487	0	1	-0.13	-0.27	-0.14 (-0.34, 0.06)	0.17

AGP, alpha-1-acid glycoprotein

*n*, 25th Percentile, and 75th Percentile are from the adjusted analyses

Adjusted for prespecified and prescreened covariates: child sex, child birth order, child gestational age, mother’s age, mother’s height, mother’s education, household food security, number of children < 18 y old in the household, number of people living in the compound, distance (in minutes) to the primary water source, household materials (wall, floor, roof), asset-based household wealth (electricity, wardrobe, table, chair or bench, khat, chouki, working radio, working black/white or color television, refrigerator, bicycle, motorcycle, sewing machine, mobile phone, land phone, number of cows, number of goats, number of chickens), and maternal exposure to intimate partner violence (IPV) during pregnancy and lifetime.

*P* < 0.2 after adjusting for multiple comparisons using the Benjamini-Hochberg procedure

### Maternal hormones and child immune status

We observed no significant associations between maternal cortisol concentration during pregnancy and child immune status at 14 and 28 mo (Table 3, Supplementary Table 4). Maternal estriol concentration was not significantly associated with child immune status at either time point. (Table 4, Supplementary Table 5).

**TABLE 3:** Maternal cortisol and child immune status.

Maternal cortisol and child immune status	Outcome	*n*	25th percentile	75th percentile	Outcome, 75th percentile vs. 25th percentile			
					Adjusted			
					Predicted outcome at 25th percentile	Predicted outcome at 75th percentile	Coefficient (95% CI)	*P*
Ln Cortisol (μg/dL)	Ln AGP Age 14 mo (g/L)	418	2.47	3.26	-0.02	-0.01	0.01 (-0.06, 0.08)	0.78
	Ln CRP Age 14 mo (mg/L)	364	2.49	3.27	-0.26	-0.09	0.16 (-0.1, 0.43)	0.23
	Ln IFN-γ Age 14 mo (pg/mL)	416	2.45	3.27	1.88	1.90	0.01 (-0.1, 0.13)	0.82
	Sum score of 13 cytokines Age 14 mo	415	2.45	3.27	-0.05	-0.05	0 (-0.24, 0.23)	0.97
	Ln AGP Age 28 mo (g/L)	250	2.48	3.24	-0.25	-0.21	0.04 (-0.07, 0.15)	0.47
	Ln CRP Age 28 mo (mg/L)	491	2.53	3.26	-0.24	-0.07	0.17 (-0.02, 0.36)	0.07
	Ln IFN-γ Age 28 mo (pg/mL)	483	2.52	3.26	1.64	1.68	0.04 (-0.09, 0.17)	0.54
	Sum score of 13 cytokines Age 28 mo	483	2.52	3.26	-0.18	-0.21	-0.03 (-0.16, 0.09)	0.62

AGP, alpha-1-acid glycoprotein

*n*, 25th Percentile, and 75th Percentile are from the adjusted analyses

Adjusted for prespecified and prescreened covariates: the covariates mentioned in Table 2 and time of day for blood collection.

*P* < 0.2 after adjusting for multiple comparisons using the Benjamini-Hochberg procedure

**TABLE 4:** Maternal estriol and child immune status.

Maternal estriol and child immune status	Outcome	*n*	25th percentile	75th percentile	Outcome, 75th percentile vs. 25th percentile			
					Adjusted			
					Predicted outcome at 25th percentile	Predicted outcome at 75th percentile	Coefficient (95% CI)	*P*
Ln Estriol (ng/mL)	Ln AGP Age 14 mo (g/L)	417	0.73	1.71	-0.05	0.00	0.06 (-0.02, 0.13)	0.16
	Ln CRP Age 14 mo (mg/L)	363	0.73	1.71	-0.23	-0.14	0.09 (-0.12, 0.31)	0.4
	Ln IFN-γ Age 14 mo (pg/mL)	405	0.76	1.71	1.98	1.98	0 (-0.07, 0.07)	0.97
	Sum score of 13 cytokines Age 14 mo	404	0.75	1.71	0.10	0.05	-0.05 (-0.41, 0.3)	0.78
	Ln AGP Age 28 mo (g/L)	250	0.70	1.70	-0.25	-0.09	0.16 (-0.1, 0.41)	0.23
	Ln CRP Age 28 mo (mg/L)	491	0.82	1.74	-0.10	0.03	0.13 (-0.03, 0.28)	0.12
	Ln IFN-γ Age 28 mo (pg/mL)	482	0.78	1.73	1.71	1.71	0 (-0.08, 0.08)	0.99
	Sum score of 13 cytokines Age 28 mo	482	0.78	1.73	-0.22	-0.17	0.05 (-0.1, 0.2)	0.54

AGP, alpha-1-acid glycoprotein

*n*, 25th Percentile, and 75th Percentile are from the adjusted analyses

Adjusted for prespecified and prescreened covariates: the covariates mentioned in Table 2.

*P* < 0.2 after adjusting for multiple comparisons using the Benjamini-Hochberg procedure

### Maternal immune status and child immune status

The positive association between maternal IFN-γ at enrollment and child IFN-γ at 14 mo was significant with a *P* value of 0.04 (0.07 log pg/mL adjusted difference [95% CI 0, 0.14]; Table 5, Supplementary Table 6). Because the other maternal immune status biomarkers were not significantly associated with child immune status biomarkers at either time point and this association was not significant after correction, this result may be spurious.

**TABLE 5:** Maternal immune status and child immune status.

Maternal immune status and child immune status	Outcome	*n*	25th percentile	75th percentile	Outcome, 75th percentile vs. 25th percentile			
					Adjusted			
					Predicted outcome at 25th percentile	Predicted outcome at 75th percentile	Coefficient (95% CI)	*P*
Ln AGP (g/L)	Ln AGP Age 14 mo (g/L)	421	-1.14	-0.54	-0.04	-0.03	0.01 (-0.07, 0.09)	0.81
	Ln CRP Age 14 mo (mg/L)	367	-1.11	-0.53	-0.16	-0.22	-0.06 (-0.24, 0.12)	0.51
	Ln IFN-γ Age 14 mo (pg/mL)	408	-1.14	-0.54	1.99	1.96	-0.03 (-0.11, 0.05)	0.46
	Sum score of 13 cytokines Age 14 mo	407	-1.14	-0.54	0.07	-0.19	-0.25 (-0.54, 0.04)	0.09
	Ln AGP Age 28 mo (g/L)	250	-1.2	-0.62	-0.1	-0.13	-0.02 (-0.21, 0.16)	0.82
	Ln CRP Age 28 mo (mg/L)	496	-1.14	-0.58	-0.01	-0.08	-0.07 (-0.24, 0.09)	0.39
	Ln IFN-γ Age 28 mo (pg/mL)	487	-1.11	-0.6	1.72	1.74	0.03 (-0.03, 0.09)	0.38
	Sum score of 13 cytokines Age 28 mo	487	-1.11	-0.6	-0.16	-0.16	0 (-0.09, 0.09)	1
Ln CRP (mg/L)	Ln AGP Age 14 mo (g/L)	421	-0.14	1.41	-0.05	-0.01	0.04 (-0.05, 0.14)	0.39
	Ln CRP Age 14 mo (mg/L)	367	-0.09	1.46	-0.12	0.11	0.24 (-0.34, 0.81)	0.43
	Ln IFN-γ Age 14 mo (pg/mL)	408	-0.13	1.38	1.98	1.99	0.01 (-0.06, 0.08)	0.8
	Sum score of 13 cytokines Age 14 mo	407	-0.13	1.38	0.04	0.04	0 (-0.13, 0.14)	0.97
	Ln AGP Age 28 mo (g/L)	250	-0.13	1.36	-0.05	-0.13	-0.09 (-0.18, 0)	0.06
	Ln CRP Age 28 mo (mg/L)	496	-0.06	1.42	-0.05	-0.06	-0.01 (-0.16, 0.15)	0.91
	Ln IFN-γ Age 28 mo (pg/mL)	487	-0.12	1.41	1.75	1.74	-0.01 (-0.11, 0.09)	0.81
	Sum score of 13 cytokines Age 28 mo	487	-0.12	1.41	-0.12	-0.22	-0.1 (-0.31, 0.12)	0.38
Ln IFN-γ (pg/mL)	Ln AGP Age 14 mo (g/L)	354	1.67	2.3	-0.05	-0.04	0.01 (-0.05, 0.06)	0.86
	Ln CRP Age 14 mo (mg/L)	307	1.66	2.29	-0.01	-0.03	-0.01 (-0.2, 0.18)	0.91
	Ln IFN-γ Age 14 mo (pg/mL)	341	1.67	2.3	1.95	2.03	0.07 (0, 0.14)	0.04
	Sum score of 13 cytokines Age 14 mo	340	1.67	2.3	0.16	0.17	0.01 (-0.21, 0.23)	0.93
	Ln AGP Age 28 mo (g/L)	247	1.56	2.14	-0.15	-0.05	0.1 (-0.05, 0.25)	0.2
	Ln CRP Age 28 mo (mg/L)	472	1.65	2.3	-0.06	-0.05	0 (-0.04, 0.04)	0.88
	Ln IFN-γ Age 28 mo (pg/mL)	460	1.64	2.3	1.81	1.81	-0.01 (-0.08, 0.06)	0.86
	Sum score of 13 cytokines Age 28 mo	460	1.64	2.3	-0.11	-0.14	-0.04 (-0.14, 0.07)	0.53
Sum score of 13 cytokines	Ln AGP Age 14 mo (g/L)	354	-0.63	0.63	-0.04	-0.02	0.02 (-0.05, 0.09)	0.54
	Ln CRP Age 14 mo (mg/L)	307	-0.66	0.61	-0.01	-0.04	-0.04 (-0.25, 0.18)	0.75
	Ln IFN-γ Age 14 mo (pg/mL)	341	-0.63	0.63	1.95	1.95	0 (-0.18, 0.17)	0.99
	Sum score of 13 cytokines Age 14 mo	340	-0.63	0.63	-0.06	-0.09	-0.03 (-0.34, 0.27)	0.84
	Ln AGP Age 28 mo (g/L)	247	-0.77	0.51	-0.11	-0.08	0.03 (-0.11, 0.17)	0.7
	Ln CRP Age 28 mo (mg/L)	472	-0.64	0.65	-0.02	-0.11	-0.09 (-0.25, 0.07)	0.28
	Ln IFN-γ Age 28 mo (pg/mL)	460	-0.65	0.64	1.82	1.8	-0.02 (-0.1, 0.05)	0.55
	Sum score of 13 cytokines Age 28 mo	460	-0.65	0.64	-0.08	-0.17	-0.09 (-0.22, 0.03)	0.15

AGP, alpha-1-acid glycoprotein

*n*, 25th Percentile, and 75th Percentile are from the adjusted analyses

Adjusted for prespecified and prescreened covariates: the covariates mentioned in Table 2.

*P* < 0.2 after adjusting for multiple comparisons using the Benjamini-Hochberg procedure

### *Post-hoc* results

Markers of vitamin A and iron status were not associated with child cytokine ratios (Supplementary Table 7). Maternal RBP concentration was negatively associated with child IFN-γ at 14 mo in the N+WSH arm but not in the control arm (N+WSH -0.03 log pg/mL adjusted difference [-0.04, -0.01], *P*-interaction = 0.01; Table 6). At 28 mo, there was no significant effect measure modification by treatment arm.

**TABLE 6:** Effect modification of maternal micronutrients and child immune status by treatment arm.

Effect modifier	Maternal micronutrients	Outcome	*n*	Modifier value	Outcome, 75th percentile vs. 25th percentile of exposure				
					Adjusted				
					Coefficient (95% CI)	*P*	FDR corrected *P* value	*P*-interaction	FDR corrected *P*-interaction
Arm	Vitamin D (nmol/L)	Ln AGP Age 14 mo (g/L)	423	Control	-0.03 (-0.11, 0.04)	0.4	0.98		
				Nutrition + WSH	0.08 (0, 0.15)	0.04	0.84	0.21	0.68
		Ln CRP Age 14 mo (mg/L)	369	Control	-0.4 (-1.09, 0.28)	0.25	0.98		
				Nutrition + WSH	-0.12 (-0.82, 0.59)	0.76	0.98	0	0
		Ln IFN-γ Age 14 mo (pg/mL)	410	Control	0.07 (-0.41, 0.54)	0.8	0.98		
				Nutrition + WSH	0.15 (-0.37, 0.66)	0.59	0.98	0.43	0.94
		Sum score of 13 cytokines Age 14 mo	409	Control	0.12 (-0.1, 0.34)	0.27	0.98		
				Nutrition + WSH	0.09 (-0.09, 0.27)	0.34	0.98	0.82	0.95
		Ln AGP Age 28 mo (g/L)	252	Control	-0.04 (-0.18, 0.11)	0.63	0.98		
				Nutrition + WSH	-0.03 (-0.17, 0.11)	0.69	0.98	0.57	0.95
		Ln CRP Age 28 mo (mg/L)	498	Control	-0.1 (-0.22, 0.03)	0.13	0.89		
				Nutrition + WSH	0 (-0.16, 0.16)	0.98	0.98	0	0
		Ln IFN-γ Age 28 mo (pg/mL)	489	Control	-0.01 (-0.11, 0.09)	0.87	0.98		
				Nutrition + WSH	0.03 (-0.05, 0.11)	0.48	0.98	0.52	0.94
		Sum score of 13 cytokines Age 28 mo	489	Control	-0.02 (-0.17, 0.13)	0.84	0.98		
				Nutrition + WSH	0 (-0.12, 0.12)	0.97	0.98	0.77	0.95
	Vitamin D deficiency	Ln AGP Age 14 mo (g/L)	423	Control	-0.16 (-0.35, 0.02)	0.09	0.85		
				Nutrition + WSH	0.1 (-0.02, 0.23)	0.11	0.85	0.78	0.95
		Ln CRP Age 14 mo (mg/L)	369	Control	-0.24 (-0.97, 0.49)	0.53	0.98		
				Nutrition + WSH	0.39 (-0.07, 0.84)	0.1	0.85	0.09	0.54
		Ln IFN-γ Age 14 mo (pg/mL)	410	Control	0.01 (-0.22, 0.24)	0.92	0.98		
				Nutrition + WSH	0.16 (0.01, 0.32)	0.04	0.84	0.28	0.81
		Sum score of 13 cytokines Age 14 mo	409	Control	0.08 (-0.36, 0.52)	0.73	0.98		
				Nutrition + WSH	0.15 (-0.14, 0.45)	0.32	0.98	0.79	0.95
		Ln AGP Age 28 mo (g/L)	252	Control	-0.05 (-0.41, 0.32)	0.8	0.98		
				Nutrition + WSH	-0.01 (-0.19, 0.17)	0.9	0.98	0.86	0.95
		Ln CRP Age 28 mo (mg/L)	498	Control	-0.27 (-0.79, 0.26)	0.32	0.98		
				Nutrition + WSH	0.22 (-0.14, 0.59)	0.23	0.98	0.13	0.6
		Ln IFN-γ Age 28 mo (pg/mL)	489	Control	-0.02 (-0.27, 0.23)	0.88	0.98		
				Nutrition + WSH	0.03 (-0.14, 0.21)	0.72	0.98	0.91	0.95
		Sum score of 13 cytokines Age 28 mo	489	Control	0.27 (-0.11, 0.65)	0.16	0.98		
				Nutrition + WSH	-0.07 (-0.33, 0.2)	0.63	0.98	0.14	0.6
	Ln RBP (μmol/L)	Ln AGP Age 14 mo (g/L)	423	Control	0 (-0.01, 0.01)	0.82	0.98		
				Nutrition + WSH	0 (-0.01, 0.01)	0.66	0.98	0.9	0.95
		Ln CRP Age 14 mo (mg/L)	369	Control	-0.01 (-0.04, 0.02)	0.61	0.98		
				Nutrition + WSH	-0.02 (-0.05, 0.01)	0.11	0.85	0.59	0.95
		Ln IFN-γ Age 14 mo (pg/mL)	410	Control	0.01 (-0.01, 0.04)	0.25	0.98		
				Nutrition + WSH	-0.03 (-0.04, -0.01)	0.01	0.39	0.01	0.13 1
		Sum score of 13 cytokines Age 14 mo	409	Control	-0.03 (-0.26, 0.2)	0.84	0.98		
				Nutrition + WSH	-0.09 (-0.32, 0.14)	0.44	0.98	0.02	0.2 1
		Ln AGP Age 28 mo (g/L)	252	Control	-0.02 (-0.11, 0.06)	0.63	0.98		
				Nutrition + WSH	0.04 (-0.01, 0.09)	0.1	0.85	0.23	0.7
		Ln CRP Age 28 mo (mg/L)	498	Control	0 (-0.01, 0.01)	0.81	0.98		
				Nutrition + WSH	0.01 (-0.01, 0.03)	0.44	0.98	0.87	0.95
		Ln IFN-γ Age 28 mo (pg/mL)	489	Control	0.01 (0, 0.02)	0.09	0.85		
				Nutrition + WSH	0.01 (0, 0.02)	0.16	0.98	0.95	0.96
		Sum score of 13 cytokines Age 28 mo	489	Control	0.02 (0, 0.04)	0.06	0.84		
				Nutrition + WSH	0.01 (-0.01, 0.02)	0.43	0.98	0.41	0.94
	Vitamin A deficiency	Ln AGP Age 14 mo (g/L)	423	Control	0.36 (-0.23, 0.96)	0.23	0.98		
				Nutrition + WSH	-0.17 (-0.66, 0.32)	0.5	0.98	0.15	0.6
		Ln CRP Age 14 mo (mg/L)	369	Control	0.02 (-0.87, 0.91)	0.97	0.98		
				Nutrition + WSH	0.04 (-1.5, 1.58)	0.96	0.98	0.11	0.54
		Ln IFN-γ Age 14 mo (pg/mL)	410	Control	0.31 (-0.44, 1.06)	0.43	0.98		
				Nutrition + WSH	-0.64 (-1.25, -0.04)	0.04	0.84	0.06	0.39
		Sum score of 13 cytokines Age 14 mo	409	Control	0.11 (-1.31, 1.53)	0.89	0.98		
				Nutrition + WSH	-1.68 (-2.81, -0.55)	0	0.39	0.04	0.31
		Ln AGP Age 28 mo (g/L)	252	Control	0.52 (-0.5, 1.55)	0.32	0.98		
				Nutrition + WSH	0.05 (-0.37, 0.48)	0.81	0.98	0.65	0.95
		Ln CRP Age 28 mo (mg/L)	498	Control	0.09 (-0.45, 0.62)	0.76	0.98		
				Nutrition + WSH	0.15 (-0.81, 1.11)	0.77	0.98	0	0
		Ln IFN-γ Age 28 mo (pg/mL)	489	Control	0.44 (-0.28, 1.17)	0.23	0.98		
				Nutrition + WSH	0.3 (-0.18, 0.78)	0.23	0.98	0.73	0.95
		Sum score of 13 cytokines Age 28 mo	489	Control	0.52 (-0.56, 1.59)	0.35	0.98		
				Nutrition + WSH	0.2 (-0.52, 0.92)	0.59	0.98	0.53	0.94
	Ln ferritin (μg/L)	Ln AGP Age 14 mo (g/L)	423	Control	0.03 (-0.02, 0.08)	0.26	0.98		
				Nutrition + WSH	0.03 (-0.02, 0.07)	0.25	0.98	0.85	0.95
		Ln CRP Age 14 mo (mg/L)	369	Control	0.04 (-0.33, 0.4)	0.85	0.98		
				Nutrition + WSH	0.05 (-0.31, 0.41)	0.8	0.98	0.44	0.94
		Ln IFN-γ Age 14 mo (pg/mL)	410	Control	0 (-0.01, 0)	0.47	0.98		
				Nutrition + WSH	-0.01 (-0.01, 0)	0.08	0.85	0.6	0.95
		Sum score of 13 cytokines Age 14 mo	409	Control	-0.01 (-0.02, 0)	0.01	0.39		
				Nutrition + WSH	-0.01 (-0.02, 0)	0.03	0.84	0.67	0.95
		Ln AGP Age 28 mo (g/L)	252	Control	-0.02 (-0.05, 0.01)	0.23	0.98		
				Nutrition + WSH	0 (-0.03, 0.02)	0.82	0.98	0.36	0.89
		Ln CRP Age 28 mo (mg/L)	498	Control	-0.01 (-0.02, 0.01)	0.55	0.98		
				Nutrition + WSH	0.01 (-0.01, 0.03)	0.58	0.98	0.18	0.63
		Ln IFN-γ Age 28 mo (pg/mL)	489	Control	0.01 (0, 0.02)	0.12	0.85		
				Nutrition + WSH	0 (-0.01, 0.02)	0.56	0.98	0.41	0.94
		Sum score of 13 cytokines Age 28 mo	489	Control	0.25 (0, 0.49)	0.05	0.84		
				Nutrition + WSH	0.23 (-0.01, 0.48)	0.06	0.84	0.1	0.54
	Ln sTfR (mg/L)	Ln AGP Age 14 mo (g/L)	423	Control	-0.02 (-0.14, 0.1)	0.78	0.98		
				Nutrition + WSH	-0.04 (-0.13, 0.04)	0.3	0.98	0.16	0.62
		Ln CRP Age 14 mo (mg/L)	369	Control	0.52 (-0.36, 1.4)	0.25	0.98		
				Nutrition + WSH	0.32 (-0.41, 1.04)	0.4	0.98	0	0
		Ln IFN-γ Age 14 mo (pg/mL)	410	Control	-0.18 (-0.45, 0.09)	0.19	0.98		
				Nutrition + WSH	-0.16 (-0.42, 0.1)	0.22	0.98	0.6	0.95
		Sum score of 13 cytokines Age 14 mo	409	Control	-0.28 (-0.8, 0.24)	0.3	0.98		
				Nutrition + WSH	-0.34 (-0.84, 0.16)	0.18	0.98	0.15	0.61
		Ln AGP Age 28 mo (g/L)	252	Control	0.01 (-0.09, 0.12)	0.83	0.98		
				Nutrition + WSH	0.07 (-0.09, 0.22)	0.4	0.98	0	0
		Ln CRP Age 28 mo (mg/L)	498	Control	-0.28 (-0.57, 0.01)	0.06	0.84		
				Nutrition + WSH	-0.15 (-0.39, 0.1)	0.24	0.98	0.06	0.39
		Ln IFN-γ Age 28 mo (pg/mL)	489	Control	0.24 (-0.73, 1.21)	0.64	0.98		
				Nutrition + WSH	0.18 (-0.69, 1.05)	0.7	0.98	0.9	0.95
		Sum score of 13 cytokines Age 28 mo	489	Control	-0.03 (-0.41, 0.36)	0.9	0.98		
				Nutrition + WSH	-0.01 (-0.28, 0.26)	0.93	0.98	0.85	0.95
	Iron deficiency	Ln AGP Age 14 mo (g/L)	423	Control	-0.05 (-0.21, 0.1)	0.51	0.98		
				Nutrition + WSH	-0.18 (-0.3, -0.05)	0.01	0.39	0.26	0.78
		Ln CRP Age 14 mo (mg/L)	369	Control	0.01 (-0.58, 0.6)	0.97	0.98		
				Nutrition + WSH	-0.42 (-0.87, 0.03)	0.06	0.85	0.27	0.78
		Ln IFN-γ Age 14 mo (pg/mL)	410	Control	-0.02 (-0.22, 0.18)	0.85	0.98		
				Nutrition + WSH	-0.04 (-0.2, 0.12)	0.65	0.98	0.97	0.97
		Sum score of 13 cytokines Age 14 mo	409	Control	-0.17 (-0.54, 0.19)	0.36	0.98		
				Nutrition + WSH	-0.04 (-0.34, 0.26)	0.79	0.98	0.2	0.68
		Ln AGP Age 28 mo (g/L)	252	Control	-0.24 (-0.55, 0.06)	0.12	0.85		
				Nutrition + WSH	-0.04 (-0.21, 0.13)	0.66	0.98	0.33	0.89
		Ln CRP Age 28 mo (mg/L)	498	Control	0.02 (-0.42, 0.46)	0.93	0.98		
				Nutrition + WSH	-0.22 (-0.58, 0.15)	0.24	0.98	0.41	0.94
		Ln IFN-γ Age 28 mo (pg/mL)	489	Control	0.11 (-0.1, 0.32)	0.33	0.98		
				Nutrition + WSH	0.09 (-0.09, 0.26)	0.34	0.98	0.89	0.95
		Sum score of 13 cytokines Age 28 mo	489	Control	0.16 (-0.15, 0.48)	0.31	0.98		
				Nutrition + WSH	0.12 (-0.14, 0.38)	0.35	0.98	0.83	0.95

AGP, alpha-1-acid glycoprotein, FDR, false discovery rate.

*n*, 25th Percentile, and 75th Percentile are from the adjusted analyses.

Adjusted for prespecified and prescreened covariates: the covariates mentioned in Table 2.

1 *P* < 0.2 after adjusting for multiple comparisons using the Benjamini-Hochberg procedure.

The post-hoc results, excluding children with recent diarrheal and respiratory illness, were similar to the prespecified main findings (Supplementary Tables 8–11). In addition, we found that maternal RBP was negatively associated with child IFN-γ at age 14 mo (−0.28 log pg/mL adjusted difference [95% CI −0.51, −0.05]; Supplementary Table 8). At age 28 mo, maternal vitamin A deficiency was associated with lower child IFN-γ with a *P* value of 0.05 (−0.45 log pg/mL adjusted difference [−0.9, 0]). Unlike the prespecified main findings, maternal vitamin A deficiency was not significantly associated with child AGP. In this post-hoc analysis, we also observed significant maternal iron status findings. Maternal ferritin was negatively associated with child AGP at age 14 mo (−0.1 log g/L adjusted difference [−0.19, −0.01]) but positively associated with child IFN-γ at age 28 mo (0.11 log pg/mL adjusted difference [95% CI 0, 0.23]). Unlike the prespecified main results, maternal sTfR was not significantly associated with any child immune marker. Of all the post-hoc results, 2 associations remained significant after FDR correction: maternal RBP and child cytokine sum scores, as well as maternal iron deficiency and child AGP, both at 14 mo (Supplementary Table 8).

There was no significant association between maternal low vitamin A status and any child immune status biomarker at either time point.

## Discussion

In this substudy of the large and well-characterized WASH Benefits Bangladesh RCT, we found that maternal vitamin A status and iron status during pregnancy were associated with child immune status at ages 14 and 28 mo. Specifically, higher maternal vitamin A concentrations during the first or second trimester of pregnancy were associated with lower inflammatory markers in children at 14 mo and higher inflammatory markers at 28 mo. Lower maternal iron concentrations were associated with higher inflammatory markers in children at 14 and 28 mo. The finding that the *in utero* environment is associated with childhood immune status is consistent with other studies, which report that perinatal factors are related to child immune function [5].

Our findings that maternal RBP was negatively associated with child cytokine sum score and maternal vitamin A deficiency was positively associated at 14 mo are consistent with known anti-inflammatory properties of vitamin A. Sufficient maternal vitamin A concentrations may promote stronger maternal immune function, which may lead to improved child immune status from *in utero* interactions between the mother and fetus. However, we observed no strong, significant associations between measures of maternal and child immune status. An alternative explanation is that maternal vitamin A may be a marker of better overall nutrition, which contributes to better child nutrition through *in utero* exposure to nutrients and breast milk. Subsequently, better overall nutritional status in the child may result in stronger immunity [[28], [29], [30]]. Specifically, a mouse study observed that retinoic acid, a metabolite of vitamin A, causes direct downregulation of IFN-γ transcription [31]. In effect measure modification analyses by treatment arm, maternal RBP during pregnancy was negatively associated with child IFN-γ in the intervention arm but not in the control arm at 14 mo. This result was consistent in the post-hoc results, only including children without recent illness. To the contrary, maternal RBP and child IFN-γ were positively associated at 28 mo, with no evidence of effect measure modification by treatment arm. When excluding children with recent illness, vitamin A deficiency was also negatively associated with IFN-γ at 28 mo. These results suggest a nuanced relationship between maternal vitamin A and child immune status, specifically IFN-γ, that may evolve over the course of childhood.

Adequate iron concentrations are important for fetal development, and maternal iron deficiency is associated with premature delivery and low birth weight [32,33]. Sufficient iron is important for immune function because neutrophils utilize iron-dependent myeloperoxidase for bactericide [34,35]. However, studies have shown that direct iron supplementation in a child can increase a child’s susceptibility to infections and gut microbiome dysbiosis [34] because microbial pathogens utilize iron to maintain metabolism and proliferate. The human host sequesters iron during infection through the mononuclear phagocyte system, resulting in lowered serum iron concentrations [36]. In our results, maternal iron deficiency was associated with higher AGP at 14 mo. Elevated maternal sTfR, or increased demand for iron due to low concentrations of available iron, was associated with higher child CRP concentrations at age 28 mo. Based on these results, we posit the following biological mechanism: infections in the mother during pregnancy lead to poor maternal iron status, which may result in poor child iron status [37] by insufficient transfer through the placenta prenatally [38]. Iron deficiency in the child would result in impaired myeloperoxidase activity, which can leave the child more susceptible to infections. Infections in the child could trigger upregulation of proinflammatory cytokines [39]. Our post-hoc analysis excluding children with recent illness supports this proposed mechanism, as higher maternal ferritin, or iron stores, was associated with lower child AGP at 14 mo. However, maternal ferritin and iron deficiency were positively associated with child IFN-γ and AGP, respectively, at 28 mo. Similar to our vitamin A results, the relationship between maternal iron and child immune status appears to change throughout time. In summary, maternal iron concentrations during pregnancy may have a noticeable, dynamic impact on iron and immune status during early childhood.

We recognize several limitations. First, the study had multiple comparisons of several maternal and child biomarkers, which increases risk of Type 1 error. To address risk of Type 1 error, we assessed the consistency of directions in point estimates, positive or negative, between related exposures (e.g., maternal RBP and vitamin A deficiency) and outcomes (e.g., child immune markers) within each category (e.g., maternal vitamin A). Additionally, we utilized the Benjamini-Hochberg method to control for FDR. No main results were significant, although only 2 post-hoc results were significant after FDR correction. Thus, the significant results observed before corrections may have been due to chance, meaning that there may be no strong causal relationships between the markers of maternal nutrition and child immune status that we measured. However, methods of correction for false discovery may be overly conservative for a study examining correlations between maternal pregnancy biomarkers and child immune status, where small to modest effect sizes are expected, especially given the long time period between when gestational biomarkers and child immune status was assessed.

Second, there was potential for selection bias because certain individuals may have been more willing to participate in the blood sample collection process compared to others. However, there was no notable difference in enrollment characteristics of those mother-child dyads included versus excluded from the study due to missing samples or other reasons (Supplementary Table 12). This observation implies there was likely minimal bias in our selection process.

Third, child AGP and CRP measurements at age 14 mo versus maternal AGP and CRP and child CRP concentrations at age 28 mo were processed in different laboratories. This limitation may have introduced additional variation in the results. Due to different timing in funding, we were unable to perform a cross-validation study between the VitMin Lab in Germany and the iccdr,b lab in Bangladesh. However, similar methods were used at both labs; thus, the results are comparable.

Fourth, water with high-iron concentrations is difficult to disinfect by chlorination, so individuals with higher iron concentrations in their drinking water can experience more infections, possibly leading to elevated proinflammatory cytokines. Therefore, the parent trial only enrolled households with low iron concentrations in drinking water (<1 mg/L on average) to maximize the effectiveness of the water chlorination intervention. Thus, our findings may not be generalizable to other households in rural Bangladesh, in which most people drink high-iron water from wells [22,40]. Another limitation is that each pregnancy biomarker was only measured once, so the data collected may not have been representative of the whole pregnancy. The third trimester of pregnancy is the most critical period for fetal accumulation of iron [41] and the highest period of fetal demand for vitamin A [42], so the first or second trimesters for maternal micronutrient measurements may have been suboptimal timing. To reduce the timing between exposure and outcome, future studies should measure micronutrient status during the third trimester and child immune status before age one to further elucidate the potential associations observed in this study.

These findings suggest that vitamin A and iron status during pregnancy may shape child immune status during the first 1 to 2 y of life. Promoting dietary diversity and multiple micronutrient supplementations, as well as preventing infection during pregnancy, may impact maternal and child immune function. Identifying early interventions aimed at optimizing the *in utero* micronutrient milieu may be a key strategy for disease interception and promotion of healthy trajectories throughout childhood.

## Funding

This study was supported by Global Development grant OPPGD759 from the Bill & Melinda Gates Foundation to the University of California, Berkeley, and by the National Institute of Allergy and Infectious Diseases of the National Institutes of Health (grant number K01AI136885 to AL)

## Conflict of Interest

All authors received funding for either salary or consulting fees through a grant from the Bill & Melinda Gates Foundation for this study. AL received funding for salary through a grant from the National Institute of Allergy and Infectious Diseases of the National Institutes of Health. The funder approved the design of the study. However, the funder played no role in data collection, analysis, interpretation, or any decisions related to publication. The corresponding author had full access to all study data and final responsibility around decision-making while submitting for publication.

## Disclaimer

The content is solely the responsibility of the authors and does not necessarily represent the official views of the National Institutes of Health. In the interest of full disclosure, Douglas Granger is the founder and chief scientific and strategy adviser at Salimetrics LLC and SalivaBio LLC, and these relationships are managed by the policies of the committees on conflict of interest at the Johns Hopkins University School of Medicine and the University of California at Irvine.

## Data Availability

Supplementary Figure and Supplementary Tables 1 to 8 are available from the Supplementary data link in the online posting of the article and the same link in the online table of contents at https://doi.org/10.1016/xxxx.20xx.xx.xxx.

## Appendix A Supplementary data

Multimedia component1 Multimedia component1
