# Supplementary material for: Micronutrient status during pregnancy is associated with child immune status in rural Bangladesh
Source: Curr Dev Nutr. 2023 Jul 4;7(8):101969. doi: 10.1016/j.cdnut.2023.101969 (PMC10407622; doi:10.1016/j.cdnut.2023.101969)
Supplement: Multimedia component1 [file mmc1.docx]

**Supplementary Methods**

*Power calculations*

We performed post-hoc power analyses using Z-tests via the pwrss.t.reg function in R. As expected for post-hoc power calculations, they show low and frequently underpowered analyses for the analyses with null or small effects, but high power for the significant estimates with large effect sizes. We note a caveat that post-hoc power calculations can yield very noisy estimates of the effect size (1).

| **Child biomarker** | **Maternal biomarker** | **Power** |
| --- | --- | --- |
| Ln CRP Age 14 months (mg/L) | Vitamin D (nmol/L) | 0.050 |
| Ln AGP Age 14 months (g/L) | Vitamin D (nmol/L) | 0.050 |
| Ln IFN-γ Age 14 months (pg/mL) | Vitamin D (nmol/L) | 0.050 |
| Sum score of 13 cytokines Age 14 months | Vitamin D (nmol/L) | 0.051 |
| Ln CRP Age 28 months (mg/L) | Vitamin D (nmol/L) | 0.050 |
| Ln AGP Age 28 months (g/L) | Vitamin D (nmol/L) | 0.050 |
| Ln IFN-γ Age 28 months (pg/mL) | Vitamin D (nmol/L) | 0.050 |
| Sum score of 28 cytokines Age 14 months | Vitamin D (nmol/L) | 0.050 |
| Ln CRP Age 14 months (mg/L) | Vitamin D deficiency | 0.982 |
| Ln AGP Age 14 months (g/L) | Vitamin D deficiency | 0.070 |
| Ln IFN-γ Age 14 months (pg/mL) | Vitamin D deficiency | 0.651 |
| Sum score of 13 cytokines Age 14 months | Vitamin D deficiency | 0.677 |
| Ln CRP Age 28 months (mg/L) | Vitamin D deficiency | 0.297 |
| Ln AGP Age 28 months (g/L) | Vitamin D deficiency | 0.061 |
| Ln IFN-γ Age 28 months (pg/mL) | Vitamin D deficiency | 0.050 |
| Sum score of 28 cytokines Age 14 months | Vitamin D deficiency | 0.193 |
| Ln CRP Age 14 months (mg/L) | Ln RBP (μmol/L) | 1.000 |
| Ln AGP Age 14 months (g/L) | Ln RBP (μmol/L) | 0.106 |
| Ln IFN-γ Age 14 months (pg/mL) | Ln RBP (μmol/L) | 0.534 |
| Sum score of 13 cytokines Age 14 months | Ln RBP (μmol/L) | 1.000 |
| Ln CRP Age 28 months (mg/L) | Ln RBP (μmol/L) | 0.956 |
| Ln AGP Age 28 months (g/L) | Ln RBP (μmol/L) | 0.415 |
| Ln IFN-γ Age 28 months (pg/mL) | Ln RBP (μmol/L) | 0.972 |
| Sum score of 28 cytokines Age 14 months | Ln RBP (μmol/L) | 0.993 |
| Ln CRP Age 14 months (mg/L) | Vitamin A deficiency | 0.946 |
| Ln AGP Age 14 months (g/L) | Vitamin A deficiency | 0.882 |
| Ln IFN-γ Age 14 months (pg/mL) | Vitamin A deficiency | 0.402 |
| Sum score of 13 cytokines Age 14 months | Vitamin A deficiency | 1.000 |
| Ln CRP Age 28 months (mg/L) | Vitamin A deficiency | 0.999 |
| Ln AGP Age 28 months (g/L) | Vitamin A deficiency | 0.725 |
| Ln IFN-γ Age 28 months (pg/mL) | Vitamin A deficiency | 0.844 |
| Sum score of 28 cytokines Age 14 months | Vitamin A deficiency | 0.998 |
| Ln CRP Age 14 months (mg/L) | Ln ferritin (μg/L) | 0.265 |
| Ln AGP Age 14 months (g/L) | Ln ferritin (μg /L) | 0.101 |
| Ln IFN-γ Age 14 months (pg/mL) | Ln ferritin (μg /L) | 0.227 |
| Sum score of 13 cytokines Age 14 months | Ln ferritin (μg /L) | 0.674 |
| Ln CRP Age 28 months (mg/L) | Ln ferritin (μg /L) | 0.064 |
| Ln AGP Age 28 months (g/L) | Ln ferritin (μg /L) | 0.090 |
| Ln IFN-γ Age 28 months (pg/mL) | Ln ferritin (μg /L) | 0.085 |
| Sum score of 28 cytokines Age 14 months | Ln ferritin (μg /L) | 0.062 |
| Ln CRP Age 14 months (mg/L) | Ln sTfR (mg/L) | 0.091 |
| Ln AGP Age 14 months (g/L) | Ln sTfR (mg/L) | 0.234 |
| Ln IFN-γ Age 14 months (pg/mL) | Ln sTfR (mg/L) | 0.057 |
| Sum score of 13 cytokines Age 14 months | Ln sTfR (mg/L) | 0.110 |
| Ln CRP Age 28 months (mg/L) | Ln sTfR (mg/L) | 1.000 |
| Ln AGP Age 28 months (g/L) | Ln sTfR (mg/L) | 0.279 |
| Ln IFN-γ Age 28 months (pg/mL) | Ln sTfR (mg/L) | 0.113 |
| Sum score of 28 cytokines Age 14 months | Ln sTfR (mg/L) | 0.050 |
| Ln CRP Age 14 months (mg/L) | Iron deficiency | 1.000 |
| Ln AGP Age 14 months (g/L) | Iron deficiency | 0.785 |
| Ln IFN-γ Age 14 months (pg/mL) | Iron deficiency | 0.092 |
| Sum score of 13 cytokines Age 14 months | Iron deficiency | 0.501 |
| Ln CRP Age 28 months (mg/L) | Iron deficiency | 0.772 |
| Ln AGP Age 28 months (g/L) | Iron deficiency | 0.270 |
| Ln IFN-γ Age 28 months (pg/mL) | Iron deficiency | 0.345 |
| Sum score of 28 cytokines Age 14 months | Iron deficiency | 0.545 |
| Ln CRP Age 14 months (mg/L) | Ln Cortisol (μg /dL) | 0.987 |
| Ln AGP Age 14 months (g/L) | Ln Cortisol (μg /dL) | 0.059 |
| Ln IFN-γ Age 14 months (pg/mL) | Ln Cortisol (μg /dL) | 0.189 |
| Sum score of 13 cytokines Age 14 months | Ln Cortisol (μg /dL) | 0.513 |
| Ln CRP Age 28 months (mg/L) | Ln Cortisol (μg /dL) | 1.000 |
| Ln AGP Age 28 months (g/L) | Ln Cortisol (μg /dL) | 0.183 |
| Ln IFN-γ Age 28 months (pg/mL) | Ln Cortisol (μg /dL) | 0.207 |
| Sum score of 28 cytokines Age 14 months | Ln Cortisol (μg /dL) | 0.530 |
| Ln CRP Age 14 months (mg/L) | Ln Estriol (ng/mL) | 0.410 |
| Ln AGP Age 14 months (g/L) | Ln Estriol (ng/mL) | 0.100 |
| Ln IFN-γ Age 14 months (pg/mL) | Ln Estriol (ng/mL) | 0.051 |
| Sum score of 13 cytokines Age 14 months | Ln Estriol (ng/mL) | 0.052 |
| Ln CRP Age 28 months (mg/L) | Ln Estriol (ng/mL) | 0.857 |
| Ln AGP Age 28 months (g/L) | Ln Estriol (ng/mL) | 0.062 |
| Ln IFN-γ Age 28 months (pg/mL) | Ln Estriol (ng/mL) | 0.052 |
| Sum score of 28 cytokines Age 14 months | Ln Estriol (ng/mL) | 0.078 |
| Ln CRP Age 14 months (mg/L) | Ln AGP (g/L) | 0.415 |
| Ln AGP Age 14 months (g/L) | Ln AGP (g/L) | 0.160 |
| Ln IFN-γ Age 14 months (pg/mL) | Ln AGP (g/L) | 0.070 |
| Sum score of 13 cytokines Age 14 months | Ln AGP (g/L) | 0.161 |
| Ln CRP Age 28 months (mg/L) | Ln AGP (g/L) | 0.932 |
| Ln AGP Age 28 months (g/L) | Ln AGP (g/L) | 0.308 |
| Ln IFN-γ Age 28 months (pg/mL) | Ln AGP (g/L) | 0.144 |
| Sum score of 28 cytokines Age 14 months | Ln AGP (g/L) | 0.051 |
| Ln CRP Age 14 months (mg/L) | Ln CRP (mg/L) | 0.085 |
| Ln AGP Age 14 months (g/L) | Ln CRP (mg/L) | 0.054 |
| Ln IFN-γ Age 14 months (pg/mL) | Ln CRP (mg/L) | 0.053 |
| Sum score of 13 cytokines Age 14 months | Ln CRP (mg/L) | 0.050 |
| Ln CRP Age 28 months (mg/L) | Ln CRP (mg/L) | 0.052 |
| Ln AGP Age 28 months (g/L) | Ln CRP (mg/L) | 0.148 |
| Ln IFN-γ Age 28 months (pg/mL) | Ln CRP (mg/L) | 0.064 |
| Sum score of 28 cytokines Age 14 months | Ln CRP (mg/L) | 0.093 |
| Ln CRP Age 14 months (mg/L) | Ln IFN-γ (pg/mL) | 0.073 |
| Ln AGP Age 14 months (g/L) | Ln IFN-γ (pg/mL) | 0.053 |
| Ln IFN-γ Age 14 months (pg/mL) | Ln IFN-γ (pg/mL) | 0.510 |
| Sum score of 13 cytokines Age 14 months | Ln IFN-γ (pg/mL) | 0.574 |
| Ln CRP Age 28 months (mg/L) | Ln IFN-γ (pg/mL) | 0.909 |
| Ln AGP Age 28 months (g/L) | Ln IFN-γ (pg/mL) | 0.114 |
| Ln IFN-γ Age 28 months (pg/mL) | Ln IFN-γ (pg/mL) | 0.109 |
| Sum score of 28 cytokines Age 14 months | Ln IFN-γ (pg/mL) | 0.176 |
| Ln CRP Age 14 months (mg/L) | Sum score of 13 cytokines | 0.095 |
| Ln AGP Age 14 months (g/L) | Sum score of 13 cytokines | 0.078 |
| Ln IFN-γ Age 14 months (pg/mL) | Sum score of 13 cytokines | 0.125 |
| Sum score of 13 cytokines Age 14 months | Sum score of 13 cytokines | 0.194 |
| Ln CRP Age 28 months (mg/L) | Sum score of 13 cytokines | 0.335 |
| Ln AGP Age 28 months (g/L) | Sum score of 13 cytokines | 0.072 |
| Ln IFN-γ Age 28 months (pg/mL) | Sum score of 13 cytokines | 0.074 |
| Sum score of 28 cytokines Age 14 months | Sum score of 13 cytokines | 0.129 |

**Supplementary Tables**

**Supplementary Table 1.** Maternal Biomarkers

|  | At Enrollment |
| --- | --- |
| Maternal Biomarker | Median (25th, 75th percentile) or n (%) |
| Vitamin D^(2)^ (nmol/L) | 42.1 (32.53, 55.19) |
| Vitamin D deficiency | 108 (19%) |
| RBP (μmol/L) | 1.24 (1.02, 1.52) |
| Vitamin A deficiency | 12 (2%) |
| Ferritin (μg/L) | 24.64 (12.89, 48.33) |
| sTfR (mg/L) | 4.28 (3.62, 5.42) |
| Iron deficiency | 134 (23%) |
| Cortisol^(3)^ (μg/dL) | 18.9 (12.36, 26.16) |
| Estriol (ng/mL) | 3.87 (2.05, 5.59) |
| Cytokine sum score | 0.12 (-0.64, 0.65) |
| IL-1β (pg/mL) | 1.12 (0.72, 1.67) |
| IL-6^(3)^ (pg/mL) | 2.29 (1.26, 3.55) |
| TNF-α (pg/mL) | 3.71 (2.93, 4.55) |
| IL-12 (pg/mL) | 2.72 (1.94, 3.67) |
| IFN-γ^(4)^ (pg/mL) | 7.27 (5.22, 10.04) |
| IL-4 (pg/mL) | 52.46 (35.58, 72.29) |
| IL-5 (pg/mL) | 1.43 (1.02, 1.99) |
| IL-13 (pg/mL) | 6.12 (3.46, 9.38) |
| IL-17A^(3)^ (pg/mL) | 4.79 (3.53, 6.49) |
| IL-21 (pg/mL) | 1.71 (0.99, 2.77) |
| IL-10^(4)^ (pg/mL) | 6.77 (3.92, 11.21) |
| IL-2 (pg/mL) | 1.04 (0.5, 1.71) |
| GM-CSF (pg/mL) | 44.82 (25.58, 71.63) |
| AGP (g/L) | 0.44 (0.33, 0.57) |
| CRP (mg/L) | 1.94 (0.91, 4.06) |

(2) Recommended vitamin D serum levels during pregnancy range from 50 to 75 nmol/L.

(3) During the first trimester of pregnancy, serum cortisol levels are expected to be 7 to 19 μg/dL, while it is expected to be around 10 to 42 μg/dL during the second trimester.

(4) A healthy population less than 45 years of age with no prior history of hypertension, diabetes, cardiovascular disease, cancer, or chronic inflammation had the following serum cytokine measurements in pg/mL: IL-6 (mean 2.91 ± standard deviation 6.45), IL-17 (6.53 ± 7.42), IL-10 (1.32 ± 3.06), and IFN-γ (13.1 ± 22.7).

**Supplementary Table 2.** Child Biomarkers

|  | Age 14 Months | Age 28 Months |
| --- | --- | --- |
| Child Biomarker | Median (25th, 75th percentile) | Median (25th, 75th percentile) |
| Cytokine Sum Score | 0.04 (-0.69, 0.63) | 0.01 (-0.66, 0.62) |
| IL-1B (pg/mL) | 1.05 (0.67, 1.53) | 1.11 (0.72, 1.59) |
| IL-6 (pg/mL) | 2.34 (1.4, 3.68) | 2.09 (1.17, 3.3) |
| TNF-α (pg/mL) | 6.27 (4.19, 8.76) | 4.64 (3.36, 6.28) |
| IL-12 (pg/mL) | 2.7 (1.93, 3.71) | 2.52 (1.71, 3.34) |
| IFN-γ (pg/mL) | 7.7 (5.43, 9.97) | 6.83 (4.99, 9.38) |
| IL-4 (pg/mL) | 45.2 (33.63, 71.53) | 44.31 (30.14, 63.26) |
| IL-5 (pg/mL) | 1.72 (1.25, 2.42) | 1.54 (1.04, 2.11) |
| IL-13 (pg/mL) | 5.64 (3.04, 9.74) | 4.97 (2.55, 8.48) |
| IL-17A (pg/mL) | 5.05 (3.47, 7.36) | 4.4 (2.82, 6.2) |
| IL-21 (pg/mL) | 1.76 (1.04, 2.69) | 1.52 (0.79, 2.26) |
| IL-10 (pg/mL) | 10.3 (6.88, 14.62) | 8.22 (5.15, 11.82) |
| IL-2 (pg/mL) | 1.05 (0.55, 1.62) | 1.03 (0.53, 1.6) |
| GM-CSF (pg/mL) | 57.27 (32.12, 98.87) | 111.15 (56.8, 205.34) |
| AGP (g/L) | 1.02 (0.77, 1.4) | N/A |
| CRP (mg/L) | 1.17 (0.39, 3.39) | 0.01 (-0.66, 0.62) |

**Supplementary Table 3.** Maternal Micronutrients and Child Immune Status

| Maternal Micronutrients and Child Immune Status | Outcome | N | 25th Percentile | 75th Percentile | Outcome, 75th Percentile v. 25th Percentile | | | | | | | | | |
| --- | --- | --- | --- | --- | --- | --- | --- | --- | --- | --- | --- | --- | --- | --- |
|  |  |  |  |  | Unadjusted | | | | | Adjusted | | | | |
|  |  |  |  |  | Predicted Outcome at 25th Percentile | Predicted Outcome at 75th Percentile | Coefficient (95% CI) | P-value | FDR Corrected P-value | Predicted Outcome at 25th Percentile | Predicted Outcome at 75th Percentile | Coefficient (95% CI) | P-value | FDR Corrected P-value |
| Vitamin D (nmol/L) | Ln AGP Age 14 months (g/L) | 434 | 32.53 | 54.57 | -0.13 | -0.09 | 0.03 (-0.02, 0.09) | 0.26 | 0.73 | -0.03 | -0.04 | -0.01 (-0.12, 0.09) | 0.84 | 0.9 |
|  | Ln CRP Age 14 months (mg/L) | 374 | 32.54 | 54.48 | 0.18 | 0.27 | 0.09 (-0.16, 0.35) | 0.48 | 0.85 | -0.23 | -0.06 | 0.17 (-0.1, 0.43) | 0.23 | 0.6 |
|  | Ln IFN-γ Age 14 months (pg/mL) | 420 | 32.16 | 54.37 | 1.91 | 1.87 | -0.04 (-0.17, 0.09) | 0.58 | 0.85 | 2.01 | 1.96 | -0.05 (-0.18, 0.08) | 0.43 | 0.69 |
|  | Sum score of 13 cytokines Age 14 months | 420 | 32.16 | 54.37 | -0.46 | -0.35 | 0.11 (-0.02, 0.23) | 0.1 | 0.44 | 0 | 0.1 | 0.1 (-0.04, 0.23) | 0.15 | 0.5 |
|  | Ln AGP Age 28 months (g/L) | 251 | 33.19 | 56.35 | -0.2 | -0.25 | -0.05 (-0.22, 0.12) | 0.58 | 0.85 | -0.2 | -0.26 | -0.06 (-0.24, 0.12) | 0.54 | 0.8 |
|  | Ln CRP Age 28 months (mg/L) | 498 | 32.59 | 55.51 | -0.11 | -0.15 | -0.04 (-0.2, 0.12) | 0.63 | 0.85 | -0.05 | -0.05 | 0.01 (-0.01, 0.02) | 0.61 | 0.8 |
|  | Ln IFN-γ Age 28 months (pg/mL) | 489 | 32.53 | 55.44 | 1.6 | 1.6 | 0 (-0.08, 0.08) | 0.99 | 1 | 1.73 | 1.75 | 0.02 (-0.06, 0.1) | 0.67 | 0.83 |
|  | Sum score of 13 cytokines Age 28 months | 489 | 32.53 | 55.44 | -0.53 | -0.57 | -0.03 (-0.15, 0.08) | 0.57 | 0.85 | -0.16 | -0.16 | -0.01 (-0.12, 0.11) | 0.93 | 0.95 |
|  |  |  |  |  |  |  |  |  |  |  |  |  |  |  |
| Vitamin D deficiency | Ln AGP Age 14 months (g/L) | 434 | 0 | 1 | -0.11 | -0.11 | 0 (-0.11, 0.1) | 0.96 | 1 | -0.02 | -0.04 | -0.02 (-0.12, 0.09) | 0.78 | 0.9 |
|  | Ln CRP Age 14 months (mg/L) | 374 | 0 | 1 | 0.22 | 0.12 | -0.1 (-0.49, 0.29) | 0.61 | 0.85 | -0.13 | -0.34 | -0.21 (-0.6, 0.18) | 0.29 | 0.63 |
|  | Ln IFN-γ Age 14 months (pg/mL) | 420 | 0 | 1 | 1.93 | 1.81 | -0.12 (-0.25, 0.01) | 0.07 | 0.44 | 2.02 | 1.9 | -0.12 (-0.25, 0.01) | 0.08 | 0.44 |
|  | Sum score of 13 cytokines Age 14 months | 420 | 0 | 1 | -0.39 | -0.48 | -0.09 (-0.33, 0.15) | 0.46 | 0.85 | 0.08 | -0.05 | -0.13 (-0.38, 0.12) | 0.3 | 0.63 |
|  | Ln AGP Age 28 months (g/L) | 251 | 0 | 1 | -0.19 | -0.15 | 0.04 (-0.12, 0.2) | 0.64 | 0.85 | -0.1 | -0.08 | 0.02 (-0.14, 0.18) | 0.82 | 0.9 |
|  | Ln CRP Age 28 months (mg/L) | 498 | 0 | 1 | -0.11 | -0.16 | -0.05 (-0.35, 0.25) | 0.76 | 0.95 | -0.04 | -0.1 | -0.06 (-0.36, 0.24) | 0.69 | 0.84 |
|  | Ln IFN­-γ Age 28 months (pg/mL) | 489 | 0 | 1 | 1.59 | 1.6 | 0.01 (-0.14, 0.15) | 0.95 | 1 | 1.74 | 1.72 | -0.02 (-0.16, 0.13) | 0.83 | 0.9 |
|  | Sum score of 13 cytokines Age 28 months | 489 | 0 | 1 | -0.54 | -0.57 | -0.03 (-0.24, 0.19) | 0.81 | 0.97 | -0.14 | -0.18 | -0.04 (-0.26, 0.17) | 0.72 | 0.85 |
|  |  |  |  |  |  |  |  |  |  |  |  |  |  |  |
| Ln RBP (μmol/L) | Ln AGP Age 14 months (g/L) | 434 | 0.13 | 0.52 | -0.11 | -0.11 | -0.01 (-0.05, 0.04) | 0.84 | 0.98 | 0.01 | -0.01 | -0.01 (-0.06, 0.04) | 0.63 | 0.8 |
|  | Ln CRP Age 14 months (mg/L) | 374 | 0.13 | 0.52 | 0.27 | 0.14 | -0.12 (-0.31, 0.06) | 0.18 | 0.62 | -0.11 | -0.25 | -0.15 (-0.33, 0.04) | 0.13 | 0.5 |
|  | Ln IFN-γ Age 14 months (pg/mL) | 420 | 0.13 | 0.52 | 1.92 | 1.9 | -0.02 (-0.09, 0.04) | 0.45 | 0.85 | 2.01 | 1.96 | -0.05 (-0.11, 0.02) | 0.17 | 0.5 |
|  | Sum score of 13 cytokines Age 14 months | 420 | 0.13 | 0.52 | -0.31 | -0.61 | -0.3 (-0.56, -0.04) | 0.02 | 0.44 | 0.12 | -0.22 | -0.34 (-0.61, -0.07) | 0.01 | 0.26 |
|  | Ln AGP Age 28 months (g/L) | 251 | 0.13 | 0.52 | -0.2 | -0.16 | 0.04 (-0.04, 0.11) | 0.33 | 0.83 | -0.09 | -0.09 | 0 (-0.01, 0.01) | 1 | 1 |
|  | Ln CRP Age 28 months (mg/L) | 498 | 0.13 | 0.52 | -0.15 | -0.1 | 0.05 (-0.09, 0.19) | 0.51 | 0.85 | -0.04 | -0.04 | 0 (0, 0) | 0.41 | 0.69 |
|  | Ln IFN-γ Age 28 months (pg/mL) | 489 | 0.12 | 0.51 | 1.56 | 1.63 | 0.07 (0.01, 0.14) | 0.03 | 0.44 | 1.67 | 1.74 | 0.07 (0.01, 0.14) | 0.03 | 0.34 |
|  | Sum score of 13 cytokines Age 28 months | 489 | 0.12 | 0.51 | -0.6 | -0.5 | 0.1 (0, 0.2) | 0.04 | 0.44 | -0.25 | -0.16 | 0.09 (-0.01, 0.19) | 0.07 | 0.44 |
|  |  |  |  |  |  |  |  |  |  |  |  |  |  |  |
| Vitamin A deficiency | Ln AGP Age 14 months (g/L) | 434 | 0 | 1 | -0.11 | -0.11 | 0 (-0.35, 0.35) | 1 | 1 | -0.03 | -0.07 | -0.04 (-0.43, 0.34) | 0.84 | 0.9 |
|  | Ln CRP Age 14 months (mg/L) | 374 | 0 | 1 | 0.2 | 0.32 | 0.12 (-1.09, 1.33) | 0.86 | 0.98 | -0.13 | -0.1 | 0.03 (-0.07, 0.13) | 0.54 | 0.8 |
|  | Ln IFN-γ Age 14 months (pg/mL) | 420 | 0 | 1 | 1.91 | 2 | 0.09 (-0.34, 0.53) | 0.68 | 0.89 | 1.98 | 2.25 | 0.27 (-0.2, 0.75) | 0.26 | 0.61 |
|  | Sum score of 13 cytokines Age 14 months | 420 | 0 | 1 | -0.4 | 0.25 | 0.65 (-0.15, 1.45) | 0.11 | 0.44 | -0.01 | 1.01 | 1.02 (0.13, 1.91) | 0.02 | 0.34 |
|  | Ln AGP Age 28 months (g/L) | 251 | 0 | 1 | -0.18 | -0.37 | -0.19 (-0.59, 0.2) | 0.34 | 0.83 | -0.09 | -0.16 | -0.07 (-0.13, -0.02) | 0.01 | 0.26 |
|  | Ln CRP Age 28 months (mg/L) | 498 | 0 | 1 | -0.12 | -0.34 | -0.22 (-1.07, 0.62) | 0.62 | 0.85 | -0.05 | -0.3 | -0.25 (-1.1, 0.6) | 0.58 | 0.8 |
|  | Ln IFN-γ Age 28 months (pg/mL) | 489 | 0 | 1 | 1.6 | 1.27 | -0.33 (-0.72, 0.07) | 0.11 | 0.44 | 1.73 | 1.39 | -0.34 (-0.74, 0.06) | 0.09 | 0.45 |
|  | Sum score of 13 cytokines Age 28 months | 489 | 0 | 1 | -0.54 | -0.91 | -0.36 (-0.96, 0.23) | 0.23 | 0.68 | -0.16 | -0.46 | -0.3 (-0.89, 0.3) | 0.33 | 0.66 |
|  |  |  |  |  |  |  |  |  |  |  |  |  |  |  |
| Ln ferritin (μg/L) | Ln AGP Age 14 months (g/L) | 434 | 2.53 | 3.91 | -0.08 | -0.13 | -0.05 (-0.12, 0.01) | 0.11 | 0.44 | -0.01 | -0.08 | -0.07 (-0.19, 0.05) | 0.27 | 0.61 |
|  | Ln CRP Age 14 months (mg/L) | 374 | 2.52 | 3.91 | 0.6 | 0.13 | -0.46 (-1.01, 0.08) | 0.09 | 0.44 | 0.15 | -0.33 | -0.47 (-1.03, 0.08) | 0.1 | 0.45 |
|  | Ln IFN-γ Age 14 months (pg/mL) | 420 | 2.53 | 3.89 | 1.95 | 1.88 | -0.07 (-0.15, 0.02) | 0.11 | 0.44 | 2.02 | 1.94 | -0.08 (-0.17, 0.01) | 0.07 | 0.44 |
|  | Sum score of 13 cytokines Age 14 months | 420 | 2.53 | 3.89 | -0.31 | -0.47 | -0.17 (-0.32, -0.01) | 0.03 | 0.44 | 0.09 | -0.07 | -0.16 (-0.32, 0) | 0.06 | 0.44 |
|  | Ln AGP Age 28 months (g/L) | 251 | 2.49 | 3.64 | -0.15 | -0.21 | -0.06 (-0.16, 0.03) | 0.21 | 0.67 | -0.07 | -0.12 | -0.04 (-0.14, 0.05) | 0.4 | 0.69 |
|  | Ln CRP Age 28 months (mg/L) | 498 | 2.54 | 3.81 | -0.14 | -0.11 | 0.03 (-0.16, 0.22) | 0.76 | 0.95 | -0.04 | -0.06 | -0.02 (-0.21, 0.17) | 0.85 | 0.9 |
|  | Ln IFN-γ Age 28 months (pg/mL) | 489 | 2.59 | 3.87 | 1.57 | 1.62 | 0.05 (-0.04, 0.14) | 0.27 | 0.73 | 1.7 | 1.77 | 0.07 (-0.02, 0.16) | 0.14 | 0.5 |
|  | Sum score of 13 cytokines Age 28 months | 489 | 2.59 | 3.87 | -0.55 | -0.55 | 0 (-0.13, 0.13) | 0.98 | 1 | -0.23 | -0.12 | 0.11 (-0.13, 0.34) | 0.38 | 0.69 |
|  |  |  |  |  |  |  |  |  |  |  |  |  |  |  |
| Ln sTfR (mg/L) | Ln AGP Age 14 months (g/L) | 434 | 1.28 | 1.68 | -0.1 | -0.12 | -0.02 (-0.07, 0.02) | 0.37 | 0.84 | -0.02 | -0.04 | -0.03 (-0.08, 0.02) | 0.26 | 0.61 |
|  | Ln CRP Age 14 months (mg/L) | 374 | 1.29 | 1.68 | 0.21 | 0.14 | -0.08 (-0.3, 0.14) | 0.5 | 0.85 | -0.13 | -0.29 | -0.16 (-0.39, 0.08) | 0.19 | 0.54 |
|  | Ln IFN-γ Age 14 months (pg/mL) | 420 | 1.28 | 1.68 | 1.84 | 1.85 | 0.01 (-0.15, 0.18) | 0.9 | 1 | 1.95 | 1.9 | -0.04 (-0.21, 0.12) | 0.63 | 0.8 |
|  | Sum score of 13 cytokines Age 14 months | 420 | 1.28 | 1.68 | -0.47 | -0.51 | -0.04 (-0.34, 0.25) | 0.78 | 0.95 | 0.02 | -0.07 | -0.09 (-0.4, 0.22) | 0.58 | 0.8 |
|  | Ln AGP Age 28 months (g/L) | 251 | 1.3 | 1.67 | -0.21 | -0.16 | 0.05 (-0.03, 0.12) | 0.21 | 0.67 | -0.11 | -0.08 | 0.03 (-0.03, 0.1) | 0.35 | 0.68 |
|  | Ln CRP Age 28 months (mg/L) | 498 | 1.3 | 1.7 | -0.18 | -0.05 | 0.12 (-0.03, 0.27) | 0.11 | 0.44 | -0.14 | 0.04 | 0.18 (0, 0.36) | 0.05 | 0.44 |
|  | Ln IFN-γ Age 28 months (pg/mL) | 489 | 1.3 | 1.68 | 1.6 | 1.59 | -0.02 (-0.08, 0.05) | 0.63 | 0.85 | 1.75 | 1.69 | -0.06 (-0.22, 0.1) | 0.45 | 0.69 |
|  | Sum score of 13 cytokines Age 28 months | 489 | 1.3 | 1.68 | -0.55 | -0.55 | 0.01 (-0.09, 0.11) | 0.91 | 1 | -0.15 | -0.16 | -0.01 (-0.11, 0.09) | 0.89 | 0.92 |
|  |  |  |  |  |  |  |  |  |  |  |  |  |  |  |
| Iron deficiency | Ln AGP Age 14 months (g/L) | 434 | 0 | 1 | -0.13 | -0.01 | 0.12 (0.03, 0.22) | 0.01 | 0.44 | -0.03 | 0.1 | 0.13 (0.04, 0.23) | 0.01 | 0.26 |
|  | Ln CRP Age 14 months (mg/L) | 374 | 0 | 1 | 0.13 | 0.44 | 0.31 (-0.05, 0.67) | 0.09 | 0.44 | -0.21 | 0.05 | 0.26 (-0.1, 0.62) | 0.15 | 0.5 |
|  | Ln IFN-γ Age 14 months (pg/mL) | 420 | 0 | 1 | 1.9 | 1.94 | 0.04 (-0.08, 0.16) | 0.54 | 0.85 | 1.98 | 2.01 | 0.03 (-0.09, 0.15) | 0.63 | 0.8 |
|  | Sum score of 13 cytokines Age 14 months | 420 | 0 | 1 | -0.42 | -0.34 | 0.08 (-0.15, 0.3) | 0.51 | 0.85 | -0.02 | 0.08 | 0.1 (-0.13, 0.33) | 0.42 | 0.69 |
|  | Ln AGP Age 28 months (g/L) | 251 | 0 | 1 | -0.21 | -0.1 | 0.12 (-0.03, 0.26) | 0.12 | 0.45 | -0.13 | -0.04 | 0.09 (-0.06, 0.24) | 0.25 | 0.61 |
|  | Ln CRP Age 28 months (mg/L) | 498 | 0 | 1 | -0.14 | -0.04 | 0.1 (-0.18, 0.38) | 0.5 | 0.85 | -0.09 | 0.03 | 0.12 (-0.16, 0.4) | 0.41 | 0.69 |
|  | Ln IFN-γ Age 28 months (pg/mL) | 489 | 0 | 1 | 1.61 | 1.54 | -0.06 (-0.19, 0.07) | 0.36 | 0.84 | 1.76 | 1.67 | -0.09 (-0.23, 0.04) | 0.17 | 0.5 |
|  | Sum score of 13 cytokines Age 28 months | 489 | 0 | 1 | -0.53 | -0.61 | -0.08 (-0.28, 0.12) | 0.46 | 0.85 | -0.13 | -0.27 | -0.14 (-0.34, 0.06) | 0.17 | 0.5 |
| N, 25th Percentile, and 75th Percentile are from the unadjusted analyses.  Adjusted for pre-specified and pre-screened covariates: child sex, child birth order, child gestational age, mother’s age, mother’s height, mother’s education, household food security, number of children < 18 years old in the household, number of people living in the compound, distance (in minutes) to the primary water source, household materials (wall, floor, roof), asset-based household wealth (electricity, wardrobe, table, chair or bench, khat, chouki, working radio, working black/white or color television, refrigerator, bicycle, motorcycle, sewing machine, mobile phone, land phone, number of cows, number of goats, number of chickens), and maternal exposure to intimate partner violence (IPV) during pregnancy and lifetime. | | | | | | | | | | | | | | |

## Supplementary Table 4. Maternal Cortisol and Child Immune Status

| Maternal Cortisol and Child Immune Status | Outcome | N | 25th Percentile | 75th Percentile | Outcome, 75th Percentile v. 25th Percentile | | | | | | | | | |
| --- | --- | --- | --- | --- | --- | --- | --- | --- | --- | --- | --- | --- | --- | --- |
|  |  |  |  |  | Unadjusted | | | | | Adjusted | | | | |
|  |  |  |  |  | Predicted Outcome at 25th Percentile | Predicted Outcome at 75th Percentile | Coefficient (95% CI) | P-value | FDR Corrected P-value | Predicted Outcome at 25th Percentile | Predicted Outcome at 75th Percentile | Coefficient (95% CI) | P-value | FDR Corrected P-value |
| Ln Cortisol (μg/dL) | Ln AGP Age 14 months (g/L) | 430 | 2.47 | 3.26 | -0.12 | -0.12 | 0 (-0.06, 0.07) | 0.98 | 0.98 | -0.02 | -0.01 | 0.01 (-0.06, 0.08) | 0.78 | 0.94 |
|  | Ln CRP Age 14 months (mg/L) | 370 | 2.49 | 3.27 | 0.19 | 0.22 | 0.03 (-0.21, 0.27) | 0.82 | 0.98 | -0.26 | -0.09 | 0.16 (-0.1, 0.43) | 0.23 | 0.94 |
|  | Ln IFN-γ Age 14 months (pg/mL) | 417 | 2.45 | 3.27 | 1.86 | 1.87 | 0.01 (-0.11, 0.13) | 0.84 | 0.98 | 1.88 | 1.90 | 0.01 (-0.1, 0.13) | 0.82 | 0.94 |
|  | Sum score of 13 cytokines Age 14 months | 417 | 2.45 | 3.27 | -0.49 | -0.51 | -0.01 (-0.25, 0.22) | 0.91 | 0.98 | -0.05 | -0.05 | 0 (-0.24, 0.23) | 0.97 | 0.97 |
|  | Ln AGP Age 28 months (g/L) | 251 | 2.48 | 3.24 | -0.21 | -0.15 | 0.06 (-0.04, 0.16) | 0.27 | 0.98 | -0.25 | -0.21 | 0.04 (-0.07, 0.15) | 0.47 | 0.94 |
|  | Ln CRP Age 28 months (mg/L) | 493 | 2.53 | 3.26 | -0.22 | -0.07 | 0.15 (-0.02, 0.32) | 0.09 | 0.70 | -0.24 | -0.07 | 0.17 (-0.02, 0.36) | 0.07 | 0.58 |
|  | Ln IFN-γ Age 28 months (pg/mL) | 484 | 2.52 | 3.26 | 1.59 | 1.56 | -0.03 (-0.11, 0.06) | 0.55 | 0.98 | 1.64 | 1.68 | 0.04 (-0.09, 0.17) | 0.54 | 0.94 |
|  | Sum score of 13 cytokines Age 28 months | 484 | 2.52 | 3.26 | -0.56 | -0.61 | -0.05 (-0.17, 0.08) | 0.47 | 0.98 | -0.18 | -0.21 | -0.03 (-0.16, 0.09) | 0.62 | 0.94 |
| N, 25th Percentile, and 75th Percentile are from the unadjusted analyses.  Adjusted for pre-specified and pre-screened covariates: the covariates mentioned in Supplementary Table 3 and time of day for blood collection. | | | | | | | | | | | | | | |

**Supplementary Table 5.** Maternal Estriol and Child Immune Status

| Maternal Estriol and Child Immune Status | Outcome | N | 25th Percentile | 75th Percentile | Outcome, 75th Percentile v. 25th Percentile | | | | | | | | | |
| --- | --- | --- | --- | --- | --- | --- | --- | --- | --- | --- | --- | --- | --- | --- |
|  |  |  |  |  | Unadjusted | | | | | Adjusted | | | | |
|  |  |  |  |  | Predicted Outcome at 25th Percentile | Predicted Outcome at 75th Percentile | Coefficient (95% CI) | P-value | FDR Corrected P-value | Predicted Outcome at 25th Percentile | Predicted Outcome at 75th Percentile | Coefficient (95% CI) | P-value | FDR Corrected P-value |
| Ln Estriol (ng/mL) | Ln AGP Age 14 months (g/L) | 430 | 0.72 | 1.71 | -0.15 | -0.12 | 0.03 (-0.04, 0.09) | 0.40 | 0.64 | -0.05 | 0.00 | 0.06 (-0.02, 0.13) | 0.16 | 0.61 |
|  | Ln CRP Age 14 months (mg/L) | 370 | 0.73 | 1.71 | 0.22 | 0.18 | -0.04 (-0.21, 0.13) | 0.66 | 0.75 | -0.23 | -0.14 | 0.09 (-0.12, 0.31) | 0.40 | 0.80 |
|  | Ln IFN-γ Age 14 months (pg/mL) | 417 | 0.75 | 1.71 | 1.87 | 1.88 | 0.01 (-0.05, 0.07) | 0.76 | 0.76 | 1.98 | 1.98 | 0 (-0.07, 0.07) | 0.97 | 0.99 |
|  | Sum score of 13 cytokines Age 14 months | 417 | 0.75 | 1.71 | -0.41 | -0.51 | -0.1 (-0.42, 0.22) | 0.55 | 0.74 | 0.10 | 0.05 | -0.05 (-0.41, 0.3) | 0.78 | 0.99 |
|  | Ln AGP Age 28 months (g/L) | 251 | 0.70 | 1.71 | -0.30 | -0.15 | 0.15 (-0.09, 0.39) | 0.21 | 0.64 | -0.25 | -0.09 | 0.16 (-0.1, 0.41) | 0.23 | 0.61 |
|  | Ln CRP Age 28 months (mg/L) | 493 | 0.81 | 1.74 | -0.17 | -0.11 | 0.06 (-0.07, 0.19) | 0.37 | 0.64 | -0.10 | 0.03 | 0.13 (-0.03, 0.28) | 0.12 | 0.61 |
|  | Ln IFN-γ Age 28 months (pg/mL) | 484 | 0.78 | 1.74 | 1.53 | 1.58 | 0.05 (-0.05, 0.15) | 0.31 | 0.64 | 1.71 | 1.71 | 0 (-0.08, 0.08) | 0.99 | 0.99 |
|  | Sum score of 13 cytokines Age 28 months | 484 | 0.78 | 1.74 | -0.71 | -0.57 | 0.14 (-0.04, 0.32) | 0.12 | 0.64 | -0.22 | -0.17 | 0.05 (-0.1, 0.2) | 0.54 | 0.86 |
| N, 25th Percentile, and 75th Percentile are from the unadjusted analyses.  Adjusted for pre-specified and pre-screened covariates: the covariates mentioned in Supplementary Table 3. | | | | | | | | | | | | | | |

**Supplementary Table 6.** Maternal Immune Status and Child Immune Status

| Maternal Immune Status and Child Immune Status | Outcome | N | 25th Percentile | 75th Percentile | Outcome, 75th Percentile v. 25th Percentile | | | | | | | | | |
| --- | --- | --- | --- | --- | --- | --- | --- | --- | --- | --- | --- | --- | --- | --- |
|  |  |  |  |  | Unadjusted | | | | | Adjusted | | | | |
|  |  |  |  |  | Predicted Outcome at 25th Percentile | Predicted Outcome at 75th Percentile | Coefficient (95% CI) | P-value | FDR Corrected P-value | Predicted Outcome at 25th Percentile | Predicted Outcome at 75th Percentile | Coefficient (95% CI) | P-value | FDR Corrected P-value |
| Ln AGP (g/L) | Ln AGP Age 14 months (g/L) | 434 | -1.14 | -0.55 | -0.1 | -0.12 | -0.02 (-0.06, 0.03) | 0.49 | 1 | -0.04 | -0.03 | 0.01 (-0.07, 0.09) | 0.81 | 1 |
|  | Ln CRP Age 14 months (mg/L) | 374 | -1.11 | -0.53 | 0.2 | 0.2 | 0 (0, 0) | 1 | 1 | -0.16 | -0.22 | -0.06 (-0.24, 0.12) | 0.51 | 1 |
|  | Ln IFN-γ Age 14 months (pg/mL) | 420 | -1.14 | -0.54 | 1.93 | 1.89 | -0.04 (-0.11, 0.03) | 0.26 | 1 | 1.99 | 1.96 | -0.03 (-0.11, 0.05) | 0.46 | 1 |
|  | Sum score of 13 cytokines Age 14 months | 420 | -1.14 | -0.54 | -0.36 | -0.44 | -0.08 (-0.22, 0.06) | 0.26 | 1 | 0.07 | -0.19 | -0.25 (-0.54, 0.04) | 0.09 | 0.91 |
|  | Ln AGP Age 28 months (g/L) | 251 | -1.19 | -0.62 | -0.13 | -0.21 | -0.07 (-0.24, 0.09) | 0.4 | 1 | -0.1 | -0.13 | -0.02 (-0.21, 0.16) | 0.82 | 1 |
|  | Ln CRP Age 28 months (mg/L) | 498 | -1.14 | -0.58 | -0.1 | -0.13 | -0.04 (-0.2, 0.13) | 0.67 | 1 | -0.01 | -0.08 | -0.07 (-0.24, 0.09) | 0.39 | 1 |
|  | Ln IFN-γ Age 28 months (pg/mL) | 489 | -1.11 | -0.6 | 1.58 | 1.6 | 0.02 (-0.04, 0.08) | 0.47 | 1 | 1.72 | 1.74 | 0.03 (-0.03, 0.09) | 0.38 | 1 |
|  | Sum score of 13 cytokines Age 28 months | 489 | -1.11 | -0.6 | -0.54 | -0.55 | -0.01 (-0.1, 0.08) | 0.86 | 1 | -0.16 | -0.16 | 0 (-0.09, 0.09) | 1 | 1 |
|  |  |  |  |  |  |  |  |  |  |  |  |  |  |  |
| Ln CRP (mg/L) | Ln AGP Age 14 months (g/L) | 434 | -0.13 | 1.41 | -0.12 | -0.1 | 0.02 (-0.04, 0.09) | 0.52 | 1 | -0.05 | -0.01 | 0.04 (-0.05, 0.14) | 0.39 | 1 |
|  | Ln CRP Age 14 months (mg/L) | 374 | -0.09 | 1.46 | 0.22 | 0.19 | -0.03 (-0.24, 0.19) | 0.82 | 1 | -0.12 | 0.11 | 0.24 (-0.34, 0.81) | 0.43 | 1 |
|  | Ln IFN-γ Age 14 months (pg/mL) | 420 | -0.12 | 1.39 | 1.91 | 1.9 | -0.01 (-0.08, 0.06) | 0.72 | 1 | 1.98 | 1.99 | 0.01 (-0.06, 0.08) | 0.8 | 1 |
|  | Sum score of 13 cytokines Age 14 months | 420 | -0.12 | 1.39 | -0.41 | -0.4 | 0.02 (-0.11, 0.14) | 0.82 | 1 | 0.04 | 0.04 | 0 (-0.13, 0.14) | 0.97 | 1 |
|  | Ln AGP Age 28 months (g/L) | 251 | -0.15 | 1.36 | -0.13 | -0.22 | -0.09 (-0.18, 0) | 0.05 | 0.87 | -0.05 | -0.13 | -0.09 (-0.18, 0) | 0.06 | 0.91 |
|  | Ln CRP Age 28 months (mg/L) | 498 | -0.08 | 1.42 | -0.11 | -0.12 | -0.01 (-0.17, 0.15) | 0.88 | 1 | -0.05 | -0.06 | -0.01 (-0.16, 0.15) | 0.91 | 1 |
|  | Ln IFN-γ Age 28 months (pg/mL) | 489 | -0.13 | 1.41 | 1.62 | 1.59 | -0.03 (-0.13, 0.08) | 0.63 | 1 | 1.75 | 1.74 | -0.01 (-0.11, 0.09) | 0.81 | 1 |
|  | Sum score of 13 cytokines Age 28 months | 489 | -0.13 | 1.41 | -0.54 | -0.55 | -0.01 (-0.13, 0.1) | 0.82 | 1 | -0.12 | -0.22 | -0.1 (-0.31, 0.12) | 0.38 | 1 |
|  |  |  |  |  |  |  |  |  |  |  |  |  |  |  |
| Ln IFN-γ (pg/mL) | Ln AGP Age 14 months (g/L) | 364 | 1.67 | 2.31 | -0.11 | -0.11 | 0 (-0.06, 0.05) | 0.97 | 1 | -0.05 | -0.04 | 0.01 (-0.05, 0.06) | 0.86 | 1 |
|  | Ln CRP Age 14 months (mg/L) | 313 | 1.66 | 2.3 | 0.17 | 0.18 | 0.01 (-0.19, 0.21) | 0.93 | 1 | -0.01 | -0.03 | -0.01 (-0.2, 0.18) | 0.91 | 1 |
|  | Ln IFN-γ Age 14 months (pg/mL) | 351 | 1.66 | 2.31 | 1.84 | 1.91 | 0.07 (0, 0.14) | 0.05 | 0.87 | 1.95 | 2.03 | 0.07 (0, 0.14) | 0.04 | 0.91 |
|  | Sum score of 13 cytokines Age 14 months | 351 | 1.66 | 2.31 | -0.41 | -0.37 | 0.04 (-0.08, 0.17) | 0.5 | 1 | 0.16 | 0.17 | 0.01 (-0.21, 0.23) | 0.93 | 1 |
|  | Ln AGP Age 28 months (g/L) | 248 | 1.56 | 2.14 | -0.24 | -0.12 | 0.12 (-0.03, 0.27) | 0.11 | 1 | -0.15 | -0.05 | 0.1 (-0.05, 0.25) | 0.2 | 1 |
|  | Ln CRP Age 28 months (mg/L) | 473 | 1.65 | 2.3 | -0.04 | -0.09 | -0.05 (-0.2, 0.1) | 0.53 | 1 | -0.06 | -0.05 | 0 (-0.04, 0.04) | 0.88 | 1 |
|  | Ln IFN-γ Age 28 months (pg/mL) | 461 | 1.64 | 2.3 | 1.65 | 1.65 | 0 (-0.06, 0.07) | 0.91 | 1 | 1.81 | 1.81 | -0.01 (-0.08, 0.06) | 0.86 | 1 |
|  | Sum score of 13 cytokines Age 28 months | 461 | 1.64 | 2.3 | -0.49 | -0.53 | -0.04 (-0.15, 0.06) | 0.46 | 1 | -0.11 | -0.14 | -0.04 (-0.14, 0.07) | 0.53 | 1 |
|  |  |  |  |  |  |  |  |  |  |  |  |  |  |  |
| Sum score of 13 cytokines | Ln AGP Age 14 months (g/L) | 364 | -0.62 | 0.64 | -0.1 | -0.1 | 0 (-0.07, 0.07) | 0.98 | 1 | -0.04 | -0.02 | 0.02 (-0.05, 0.09) | 0.54 | 1 |
|  | Ln CRP Age 14 months (mg/L) | 313 | -0.65 | 0.62 | 0.2 | 0.15 | -0.05 (-0.27, 0.17) | 0.67 | 1 | -0.01 | -0.04 | -0.04 (-0.25, 0.18) | 0.75 | 1 |
|  | Ln IFN-γ Age 14 months (pg/mL) | 351 | -0.63 | 0.65 | 1.84 | 1.84 | -0.01 (-0.18, 0.16) | 0.94 | 1 | 1.95 | 1.95 | 0 (-0.18, 0.17) | 0.99 | 1 |
|  | Sum score of 13 cytokines Age 14 months | 351 | -0.63 | 0.65 | -0.4 | -0.46 | -0.06 (-0.34, 0.22) | 0.69 | 1 | -0.06 | -0.09 | -0.03 (-0.34, 0.27) | 0.84 | 1 |
|  | Ln AGP Age 28 months (g/L) | 248 | -0.79 | 0.51 | -0.21 | -0.16 | 0.05 (-0.09, 0.18) | 0.51 | 1 | -0.11 | -0.08 | 0.03 (-0.11, 0.17) | 0.7 | 1 |
|  | Ln CRP Age 28 months (mg/L) | 473 | -0.65 | 0.65 | -0.06 | -0.11 | -0.05 (-0.21, 0.11) | 0.57 | 1 | -0.02 | -0.11 | -0.09 (-0.25, 0.07) | 0.28 | 1 |
|  | Ln IFN-γ Age 28 months (pg/mL) | 461 | -0.66 | 0.64 | 1.65 | 1.65 | -0.01 (-0.08, 0.06) | 0.86 | 1 | 1.82 | 1.8 | -0.02 (-0.1, 0.05) | 0.55 | 1 |
|  | Sum score of 13 cytokines Age 28 months | 461 | -0.66 | 0.64 | -0.45 | -0.55 | -0.09 (-0.22, 0.03) | 0.15 | 1 | -0.08 | -0.17 | -0.09 (-0.22, 0.03) | 0.15 | 1 |
| N, 25th Percentile, and 75th Percentile are from the unadjusted analyses.  Adjusted for pre-specified and pre-screened covariates: the covariates mentioned in Supplementary Table 3. | | | | | | | | | | | | | | |

**Supplementary Table 7.** Maternal RBP and sTfR and Child Cytokine Ratios

| Maternal RBP and sTfR and Child Cytokine Ratios | Outcome | N | 25th Percentile | 75th Percentile | Outcome, 75th Percentile v. 25th Percentile | | | | | | | | | |
| --- | --- | --- | --- | --- | --- | --- | --- | --- | --- | --- | --- | --- | --- | --- |
|  |  |  |  |  | Unadjusted | | | | | Adjusted | | | | |
|  |  |  |  |  | Predicted Outcome at 25th Percentile | Predicted Outcome at 75th Percentile | Coefficient (95% CI) | P-value | FDR Corrected P-value | Predicted Outcome at 25th Percentile | Predicted Outcome at 75th Percentile | Coefficient (95% CI) | P-value | FDR Corrected P-value |
| Ln RBP (μmol/L) | Th1/Th2 Age 14 months | 415 | 0.13 | 0.52 | 0.08 | 0.09 | 0.01 (-0.05, 0.06) | 0.81 | 0.9 | 0.07 | 0.04 | -0.03 (-0.08, 0.03) | 0.32 | 0.78 |
|  | Th1/IL-10 Age 14 months | 417 | 0.13 | 0.52 | 1.07 | 1.21 | 0.13 (-0.06, 0.32) | 0.18 | 0.65 | 0.95 | 1.05 | 0.09 (-0.1, 0.29) | 0.34 | 0.78 |
|  | Th2/IL-10 Age 14 months | 415 | 0.13 | 0.52 | 0.95 | 0.97 | 0.02 (-0.2, 0.24) | 0.86 | 0.9 | 0.82 | 0.84 | 0.02 (-0.19, 0.24) | 0.84 | 0.91 |
|  | Proinflammatory/IL-10 Age 14 months | 416 | 0.13 | 0.52 | 1.21 | 1.43 | 0.22 (0, 0.44) | 0.05 | 0.51 | 1.17 | 1.37 | 0.21 (-0.02, 0.43) | 0.07 | 0.59 |
|  | Th1/Th2 Age 28 months | 488 | 0.12 | 0.51 | -0.2 | -0.21 | -0.01 (-0.06, 0.05) | 0.8 | 0.9 | -0.11 | -0.12 | -0.01 (-0.07, 0.05) | 0.75 | 0.91 |
|  | Th1/IL-10 Age 28 months | 485 | 0.12 | 0.51 | 1.42 | 1.39 | -0.03 (-0.09, 0.03) | 0.34 | 0.79 | 1.38 | 1.36 | -0.02 (-0.09, 0.04) | 0.44 | 0.82 |
|  | Th2/IL-10 Age 28 months | 484 | 0.12 | 0.51 | 1.56 | 1.54 | -0.02 (-0.09, 0.05) | 0.63 | 0.9 | 1.37 | 1.36 | -0.01 (-0.09, 0.06) | 0.79 | 0.91 |
|  | Proinflammatory/IL-10 Age 28 months | 483 | 0.13 | 0.51 | 1.99 | 1.99 | -0.01 (-0.08, 0.07) | 0.9 | 0.9 | 1.72 | 1.72 | 0 (-0.07, 0.07) | 0.98 | 0.98 |
| Ln sTfR (mg/L) | Th1/Th2 Age 14 months | 415 | 1.28 | 1.68 | 0.09 | 0.08 | -0.01 (-0.06, 0.04) | 0.69 | 0.9 | 0.04 | 0.02 | -0.02 (-0.07, 0.03) | 0.46 | 0.82 |
|  | Th1/IL-10 Age 14 months | 417 | 1.28 | 1.68 | 1.12 | 1.07 | -0.05 (-0.12, 0.01) | 0.13 | 0.65 | 1.03 | 0.96 | -0.06 (-0.13, 0.01) | 0.07 | 0.59 |
|  | Th2/IL-10 Age 14 months | 415 | 1.28 | 1.68 | 0.93 | 0.9 | -0.03 (-0.1, 0.05) | 0.54 | 0.9 | 0.82 | 0.8 | -0.01 (-0.09, 0.07) | 0.78 | 0.91 |
|  | Proinflammatory/IL-10 Age 14 months | 416 | 1.28 | 1.68 | 1.34 | 1.29 | -0.04 (-0.12, 0.04) | 0.33 | 0.79 | 1.2 | 1.17 | -0.03 (-0.22, 0.16) | 0.79 | 0.91 |
|  | Th1/Th2 Age 28 months | 488 | 1.3 | 1.69 | -0.19 | -0.22 | -0.02 (-0.08, 0.04) | 0.45 | 0.9 | -0.1 | -0.13 | -0.03 (-0.09, 0.03) | 0.33 | 0.78 |
|  | Th1/IL-10 Age 28 months | 485 | 1.3 | 1.68 | 1.44 | 1.27 | -0.17 (-0.34, 0.01) | 0.06 | 0.51 | 1.4 | 1.27 | -0.13 (-0.3, 0.04) | 0.14 | 0.77 |
|  | Th2/IL-10 Age 28 months | 484 | 1.3 | 1.68 | 1.61 | 1.48 | -0.14 (-0.35, 0.07) | 0.2 | 0.65 | 1.4 | 1.28 | -0.13 (-0.34, 0.08) | 0.24 | 0.78 |
|  | Proinflammatory/IL-10 Age 28 months | 483 | 1.29 | 1.68 | 1.98 | 2 | 0.02 (-0.16, 0.2) | 0.86 | 0.9 | 1.69 | 1.71 | 0.02 (-0.17, 0.21) | 0.85 | 0.91 |

| N, 25th Percentile, and 75th Percentile are from the unadjusted analyses.  Adjusted for pre-specified and pre-screened covariates: the covariates mentioned in Supplementary Table 3. |
| --- |

**Supplementary Table 8.** Maternal Micronutrients and Child Immune Status in Children Without Recent Illness

| Maternal Micronutrients and Child Immune Status | Outcome | N | 25th Percentile | 75th Percentile | Outcome, 75th Percentile v. 25th Percentile | | | |
| --- | --- | --- | --- | --- | --- | --- | --- | --- |
|  |  |  |  |  | Adjusted | | | |
|  |  |  |  |  | Predicted Outcome at 25th Percentile | Predicted Outcome at 75th Percentile | Coefficient (95% CI) | P-value |
| Vitamin D (nmol/L) | Ln AGP Age 14 months (g/L) | 257 | 32.1 | 52.54 | -0.09 | -0.02 | 0.06 (0, 0.13) | 0.06 |
|  | Ln CRP Age 14 months (mg/L) | 232 | 32.04 | 52.62 | -0.24 | -0.04 | 0.2 (-0.11, 0.5) | 0.2 |
|  | Ln IFN- γ Age 14 months (pg/mL) | 252 | 31.66 | 51.71 | 1.65 | 1.55 | -0.1 (-0.27, 0.06) | 0.22 |
|  | Sum score of 13 cytokines Age 14 months | 252 | 31.66 | 51.71 | -0.3 | -0.51 | -0.21 (-0.48, 0.07) | 0.14 |
|  | Ln AGP Age 28 months (g/L) | 164 | 32.57 | 56.33 | -0.33 | -0.42 | -0.08 (-0.29, 0.12) | 0.43 |
|  | Ln CRP Age 28 months (mg/L) | 346 | 32.61 | 55.8 | -0.15 | -0.22 | -0.06 (-0.47, 0.34) | 0.77 |
|  | Ln IFN-γ Age 28 months (pg/mL) | 337 | 32.58 | 55.76 | 1.67 | 1.66 | -0.01 (-0.11, 0.08) | 0.84 |
|  | Sum score of 13 cytokines Age 28 months | 337 | 32.58 | 55.76 | -0.49 | -0.5 | -0.02 (-0.17, 0.13) | 0.84 |
|  |  |  |  |  |  |  |  |  |
| Vitamin D deficiency | Ln AGP Age 14 months (g/L) | 257 | 0 | 1 | -0.03 | -0.14 | -0.12 (-0.25, 0.02) | 0.09 |
|  | Ln CRP Age 14 months (mg/L) | 232 | 0 | 1 | -0.14 | -0.42 | -0.28 (-0.74, 0.18) | 0.23 |
|  | Ln IFN-γ Age 14 months (pg/mL) | 252 | 0 | 1 | 1.64 | 1.54 | -0.1 (-0.27, 0.06) | 0.22 |
|  | Sum score of 13 cytokines Age 14 months | 252 | 0 | 1 | -0.31 | -0.4 | -0.09 (-0.39, 0.21) | 0.57 |
|  | Ln AGP Age 28 months (g/L) | 164 | 0 | 1 | -0.29 | -0.36 | -0.07 (-0.25, 0.11) | 0.45 |
|  | Ln CRP Age 28 months (mg/L) | 346 | 0 | 1 | -0.27 | -0.51 | -0.25 (-0.58, 0.09) | 0.15 |
|  | Ln IFN-γ Age 28 months (pg/mL) | 337 | 0 | 1 | 1.66 | 1.7 | 0.04 (-0.14, 0.22) | 0.69 |
|  | Sum score of 13 cytokines Age 28 months | 337 | 0 | 1 | -0.5 | -0.44 | 0.07 (-0.2, 0.34) | 0.62 |
|  |  |  |  |  |  |  |  |  |
| Ln RBP (μmol/L) | Ln AGP Age 14 months (g/L) | 257 | 0.14 | 0.53 | -0.07 | -0.08 | -0.01 (-0.08, 0.05) | 0.7 |
|  | Ln CRP Age 14 months (mg/L) | 232 | 0.14 | 0.55 | 0.17 | -0.02 | -0.19 (-0.76, 0.37) | 0.51 |
|  | Ln IFN-γ Age 14 months (pg/mL) | 252 | 0.14 | 0.54 | 1.71 | 1.43 | -0.28 (-0.51, -0.05) | 0.02 |
|  | Sum score of 13 cytokines Age 14 months | 252 | 0.14 | 0.54 | -0.28 | -0.85 | -0.57 (-0.98, -0.17) | 0.01* |
|  | Ln AGP Age 28 months (g/L) | 164 | 0.13 | 0.49 | -0.31 | -0.31 | 0 (-0.07, 0.08) | 0.99 |
|  | Ln CRP Age 28 months (mg/L) | 346 | 0.13 | 0.52 | -0.42 | -0.42 | 0 (0, 0) | 0.63 |
|  | Ln IFN-γ Age 28 months (pg/mL) | 337 | 0.13 | 0.51 | 1.58 | 1.66 | 0.09 (0.01, 0.17) | 0.03 |
|  | Sum score of 13 cytokines Age 28 months | 337 | 0.13 | 0.51 | -0.61 | -0.49 | 0.12 (0.01, 0.24) | 0.04 |
|  |  |  |  |  |  |  |  |  |
| Vitamin A deficiency | Ln AGP Age 14 months (g/L) | 257 | 0 | 1 | -0.08 | -0.14 | -0.07 (-0.56, 0.43) | 0.81 |
|  | Ln CRP Age 14 months (mg/L) | 232 | 0 | 1 | -0.22 | 0.27 | 0.49 (-1.15, 2.14) | 0.57 |
|  | Ln IFN-γ Age 14 months (pg/mL) | 252 | 0 | 1 | 1.62 | 1.76 | 0.13 (-0.5, 0.76) | 0.69 |
|  | Sum score of 13 cytokines Age 14 months | 252 | 0 | 1 | -0.34 | 0.01 | 0.34 (-0.77, 1.45) | 0.56 |
|  | Ln AGP Age 28 months (g/L) | 164 | 0 | 1 | -0.31 | -0.36 | -0.05 (-0.47, 0.36) | 0.82 |
|  | Ln CRP Age 28 months (mg/L) | 346 | 0 | 1 | -0.32 | -0.69 | -0.37 (-1.25, 0.51) | 0.41 |
|  | Ln IFN-γ Age 28 months (pg/mL) | 337 | 0 | 1 | 1.66 | 1.21 | -0.45 (-0.9, 0) | 0.05 |
|  | Sum score of 13 cytokines Age 28 months | 337 | 0 | 1 | -0.49 | -0.86 | -0.38 (-1.05, 0.3) | 0.28 |
|  |  |  |  |  |  |  |  |  |
| Ln ferritin (μg/L) | Ln AGP Age 14 months (g/L) | 257 | 2.59 | 3.97 | -0.05 | -0.15 | -0.1 (-0.19, -0.01) | 0.03 |
|  | Ln CRP Age 14 months (mg/L) | 232 | 2.52 | 4.01 | -0.15 | -0.09 | 0.06 (-0.45, 0.57) | 0.84 |
|  | Ln IFN-γ Age 14 months (pg/mL) | 252 | 2.58 | 4 | 1.68 | 1.58 | -0.1 (-0.22, 0.02) | 0.09 |
|  | Sum score of 13 cytokines Age 14 months | 252 | 2.58 | 4 | -0.18 | -0.33 | -0.15 (-0.36, 0.06) | 0.17 |
|  | Ln AGP Age 28 months (g/L) | 164 | 2.56 | 3.74 | -0.31 | -0.3 | 0.01 (-0.11, 0.13) | 0.91 |
|  | Ln CRP Age 28 months (mg/L) | 346 | 2.59 | 3.89 | -0.35 | -0.31 | 0.03 (-0.15, 0.21) | 0.73 |
|  | Ln IFN-γ Age 28 months (pg/mL) | 337 | 2.61 | 3.91 | 1.61 | 1.72 | 0.11 (0, 0.23) | 0.05 |
|  | Sum score of 13 cytokines Age 28 months | 337 | 2.61 | 3.91 | -0.51 | -0.4 | 0.12 (-0.05, 0.29) | 0.18 |
|  |  |  |  |  |  |  |  |  |
| Ln sTfR (mg/L) | Ln AGP Age 14 months (g/L) | 257 | 1.27 | 1.68 | -0.09 | -0.12 | -0.03 (-0.18, 0.11) | 0.69 |
|  | Ln CRP Age 14 months (mg/L) | 232 | 1.3 | 1.7 | -0.21 | -0.28 | -0.08 (-0.31, 0.16) | 0.54 |
|  | Ln IFN-γ Age 14 months (pg/mL) | 252 | 1.27 | 1.69 | 1.64 | 1.57 | -0.07 (-0.27, 0.14) | 0.52 |
|  | Sum score of 13 cytokines Age 14 months | 252 | 1.27 | 1.69 | -0.21 | -0.27 | -0.06 (-0.43, 0.3) | 0.74 |
|  | Ln AGP Age 28 months (g/L) | 164 | 1.27 | 1.66 | -0.31 | -0.31 | 0 (-0.07, 0.08) | 0.95 |
|  | Ln CRP Age 28 months (mg/L) | 346 | 1.28 | 1.7 | -0.35 | -0.36 | -0.01 (-0.02, 0) | 0.08 |
|  | Ln IFN-γ Age 28 months (pg/mL) | 337 | 1.28 | 1.69 | 1.68 | 1.66 | -0.03 (-0.11, 0.06) | 0.55 |
|  | Sum score of 13 cytokines Age 28 months | 337 | 1.28 | 1.69 | -0.48 | -0.48 | 0 (-0.13, 0.12) | 0.97 |
|  |  |  |  |  |  |  |  |  |
| Iron deficiency | Ln AGP Age 14 months (g/L) | 257 | 0 | 1 | -0.06 | 0.15 | 0.21 (0.09, 0.33) | 0* |
|  | Ln CRP Age 14 months (mg/L) | 232 | 0 | 1 | -0.23 | 0.05 | 0.28 (-0.15, 0.72) | 0.2 |
|  | Ln IFN-γ Age 14 months (pg/mL) | 252 | 0 | 1 | 1.61 | 1.65 | 0.04 (-0.12, 0.2) | 0.65 |
|  | Sum score of 13 cytokines Age 14 months | 252 | 0 | 1 | -0.33 | -0.26 | 0.07 (-0.22, 0.36) | 0.65 |
|  | Ln AGP Age 28 months (g/L) | 164 | 0 | 1 | -0.35 | -0.26 | 0.09 (-0.09, 0.27) | 0.34 |
|  | Ln CRP Age 28 months (mg/L) | 346 | 0 | 1 | -0.32 | -0.34 | -0.02 (-0.34, 0.3) | 0.9 |
|  | Ln IFN-γ Age 28 months (pg/mL) | 337 | 0 | 1 | 1.69 | 1.57 | -0.12 (-0.29, 0.05) | 0.16 |
|  | Sum score of 13 cytokines Age 28 months | 337 | 0 | 1 | -0.45 | -0.64 | -0.2 (-0.45, 0.05) | 0.12 |
| N, 25th Percentile, and 75th Percentile are from the adjusted analyses  Adjusted for pre-specified and pre-screened covariates: the covariates mentioned in Supplementary Table 3.  Excludes children who had caregiver-reported recent illness in the past 7 days before sample collection. | | | | | | | | |
| * P-value < 0.2 after adjusting for multiple comparisons using the Benjamini-Hochberg procedure | | | | | | | | |

**Supplementary Table 9.** Maternal Cortisol and Child Immune Status in Children Without Recent Illness

| Maternal Cortisol and Child Immune Status | Outcome | N | 25th Percentile | 75th Percentile | Outcome, 75th Percentile v. 25th Percentile | | | |
| --- | --- | --- | --- | --- | --- | --- | --- | --- |
|  |  |  |  |  | Adjusted | | | |
|  |  |  |  |  | Predicted Outcome at 25th Percentile | Predicted Outcome at 75th Percentile | Coefficient (95% CI) | P-value |
| Ln Cortisol (μg/dL) | Ln AGP Age 14 months (g/L) | 259 | 2.57 | 3.28 | -0.09 | -0.12 | -0.02 (-0.11, 0.06) | 0.58 |
|  | Ln CRP Age 14 months (mg/L) | 232 | 2.61 | 3.29 | -0.24 | -0.38 | -0.14 (-0.4, 0.12) | 0.29 |
|  | Ln IFN-γ Age 14 months (pg/mL) | 255 | 2.54 | 3.28 | 1.55 | 1.54 | -0.01 (-0.11, 0.09) | 0.87 |
|  | Sum score of 13 cytokines Age 14 months | 249 | 2.57 | 3.27 | -0.39 | -0.38 | 0.01 (-0.2, 0.22) | 0.95 |
|  | Ln AGP Age 28 months (g/L) | 164 | 2.45 | 3.24 | -0.36 | -0.34 | 0.02 (-0.1, 0.14) | 0.73 |
|  | Ln CRP Age 28 months (mg/L) | 343 | 2.57 | 3.27 | -0.38 | -0.33 | 0.05 (-0.14, 0.24) | 0.63 |
|  | Ln IFN-γ Age 28 months (pg/mL) | 334 | 2.56 | 3.27 | 1.48 | 1.60 | 0.12 (-0.06, 0.31) | 0.19 |
|  | Sum score of 13 cytokines Age 28 months | 334 | 2.56 | 3.27 | -0.41 | -0.51 | -0.1 (-0.25, 0.05) | 0.21 |
| N, 25th Percentile, and 75th Percentile are from the adjusted analyses  Adjusted for pre-specified and pre-screened covariates: the covariates mentioned in Supplementary Table 3.  Excludes children who had caregiver-reported recent illness in the past 7 days before sample collection. | | | | | | | | |
|  | | | | | | | | |

**Supplementary Table 10.** Maternal Estriol and Child Immune Status in Children Without Recent Illness

| Maternal Estriol and Child Immune Status | Outcome | N | 25th Percentile | 75th Percentile | Outcome, 75th Percentile v. 25th Percentile | | | |
| --- | --- | --- | --- | --- | --- | --- | --- | --- |
|  |  |  |  |  | Adjusted | | | |
|  |  |  |  |  | Predicted Outcome at 25th Percentile | Predicted Outcome at 75th Percentile | Coefficient (95% CI) | P-value |
| Ln Estriol (ng/mL) | Ln AGP Age 14 months (g/L) | 254 | 0.78 | 1.71 | -0.14 | -0.05 | 0.09 (-0.01, 0.2) | 0.09 |
|  | Ln CRP Age 14 months (mg/L) | 229 | 0.79 | 1.71 | -0.33 | -0.35 | -0.02 (-0.29, 0.25) | 0.9 |
|  | Ln IFN-γ Age 14 months (pg/mL) | 250 | 0.79 | 1.72 | 1.60 | 1.62 | 0.02 (-0.08, 0.12) | 0.7 |
|  | Sum score of 13 cytokines Age 14 months | 250 | 0.79 | 1.72 | -0.31 | -0.21 | 0.1 (-0.34, 0.54) | 0.66 |
|  | Ln AGP Age 28 months (g/L) | 164 | 0.45 | 1.70 | -0.31 | -0.33 | -0.02 (-0.15, 0.11) | 0.73 |
|  | Ln CRP Age 28 months (mg/L) | 343 | 0.90 | 1.78 | -0.35 | -0.23 | 0.13 (-0.04, 0.3) | 0.13 |
|  | Ln IFN-γ Age 28 months (pg/mL) | 334 | 0.86 | 1.77 | 1.65 | 1.65 | 0 (-0.09, 0.09) | 1 |
|  | Sum score of 13 cytokines Age 28 months | 334 | 0.86 | 1.77 | -0.48 | -0.50 | -0.02 (-0.16, 0.12) | 0.79 |
| N, 25th Percentile, and 75th Percentile are from the adjusted analyses  Adjusted for pre-specified and pre-screened covariates: the covariates mentioned in Supplementary Table 3.  Excludes children who had caregiver-reported recent illness in the past 7 days before sample collection. | | | | | | | | |
|  | | | | | | | | |

**Supplementary Table 11.** Maternal Immune Status and Child Immune Status in Children Without Recent Illness

| Maternal Immune Status and Child Immune Status | Outcome | N | 25th Percentile | 75th Percentile | Outcome, 75th Percentile v. 25th Percentile | | | |
| --- | --- | --- | --- | --- | --- | --- | --- | --- |
|  |  |  |  |  | Adjusted | | | |
|  |  |  |  |  | Predicted Outcome at 25th Percentile | Predicted Outcome at 75th Percentile | Coefficient (95% CI) | P-value |
| Ln AGP (g/L) | Ln AGP Age 14 months (g/L) | 257 | -1.08 | -0.53 | -0.03 | -0.07 | -0.04 (-0.1, 0.02) | 0.24 |
|  | Ln CRP Age 14 months (mg/L) | 232 | -1.06 | -0.53 | -0.23 | -0.19 | 0.04 (-0.16, 0.24) | 0.73 |
|  | Ln IFN-γ Age 14 months (pg/mL) | 252 | -1.06 | -0.53 | 1.63 | 1.62 | -0.01 (-0.09, 0.07) | 0.81 |
|  | Sum score of 13 cytokines Age 14 months | 252 | -1.06 | -0.53 | -0.3 | -0.34 | -0.04 (-0.19, 0.1) | 0.58 |
|  | Ln AGP Age 28 months (g/L) | 164 | -1.18 | -0.59 | -0.3 | -0.32 | -0.02 (-0.1, 0.06) | 0.71 |
|  | Ln CRP Age 28 months (mg/L) | 346 | -1.1 | -0.56 | -0.27 | -0.34 | -0.07 (-0.24, 0.11) | 0.46 |
|  | Ln IFN-γ Age 28 months (pg/mL) | 337 | -1.08 | -0.56 | 1.64 | 1.67 | 0.03 (-0.04, 0.11) | 0.41 |
|  | Sum score of 13 cytokines Age 28 months | 337 | -1.08 | -0.56 | -0.52 | -0.47 | 0.05 (-0.07, 0.16) | 0.43 |
|  |  |  |  |  |  |  |  |  |
| Ln CRP (mg/L) | Ln AGP Age 14 months (g/L) | 257 | 0.02 | 1.49 | -0.08 | -0.13 | -0.06 (-0.14, 0.03) | 0.21 |
|  | Ln CRP Age 14 months (mg/L) | 232 | 0.07 | 1.53 | -0.19 | -0.26 | -0.07 (-0.31, 0.18) | 0.6 |
|  | Ln IFN-γ Age 14 months (pg/mL) | 252 | 0.03 | 1.46 | 1.62 | 1.61 | -0.01 (-0.11, 0.08) | 0.78 |
|  | Sum score of 13 cytokines Age 14 months | 252 | 0.03 | 1.46 | -0.32 | -0.34 | -0.02 (-0.18, 0.14) | 0.85 |
|  | Ln AGP Age 28 months (g/L) | 164 | -0.17 | 1.41 | -0.29 | -0.35 | -0.05 (-0.16, 0.05) | 0.31 |
|  | Ln CRP Age 28 months (mg/L) | 346 | -0.12 | 1.43 | -0.36 | -0.28 | 0.08 (-0.12, 0.28) | 0.45 |
|  | Ln IFN-γ Age 28 months (pg/mL) | 337 | -0.15 | 1.42 | 1.65 | 1.66 | 0.01 (-0.11, 0.13) | 0.88 |
|  | Sum score of 13 cytokines Age 28 months | 337 | -0.15 | 1.42 | -0.62 | -0.55 | 0.07 (-0.27, 0.41) | 0.69 |
|  |  |  |  |  |  |  |  |  |
| Ln IFN-γ (pg/mL) | Ln AGP Age 14 months (g/L) | 212 | 1.67 | 2.32 | -0.1 | -0.09 | 0.01 (0, 0.02) | 0.1 |
|  | Ln CRP Age 14 months (mg/L) | 192 | 1.67 | 2.31 | -0.28 | -0.3 | -0.02 (-0.25, 0.22) | 0.9 |
|  | Ln IFN-γ Age 14 months (pg/mL) | 206 | 1.66 | 2.3 | 1.66 | 1.68 | 0.02 (-0.07, 0.11) | 0.69 |
|  | Sum score of 13 cytokines Age 14 months | 206 | 1.66 | 2.3 | -0.27 | -0.32 | -0.05 (-0.26, 0.17) | 0.7 |
|  | Ln AGP Age 28 months (g/L) | 164 | 1.51 | 2.1 | -0.34 | -0.3 | 0.04 (-0.05, 0.12) | 0.38 |
|  | Ln CRP Age 28 months (mg/L) | 333 | 1.63 | 2.3 | -0.27 | -0.35 | -0.09 (-0.3, 0.13) | 0.45 |
|  | Ln IFN-γ Age 28 months (pg/mL) | 322 | 1.62 | 2.3 | 1.71 | 1.7 | -0.01 (-0.1, 0.08) | 0.86 |
|  | Sum score of 13 cytokines Age 28 months | 322 | 1.62 | 2.3 | -0.41 | -0.44 | -0.04 (-0.17, 0.1) | 0.61 |
|  |  |  |  |  |  |  |  |  |
| Sum score of 13 cytokines | Ln AGP Age 14 months (g/L) | 212 | -0.71 | 0.62 | -0.1 | -0.08 | 0.03 (-0.05, 0.1) | 0.51 |
|  | Ln CRP Age 14 months (mg/L) | 192 | -0.74 | 0.62 | -0.26 | -0.31 | -0.05 (-0.33, 0.23) | 0.76 |
|  | Ln IFN-γ Age 14 months (pg/mL) | 206 | -0.73 | 0.62 | 1.67 | 1.66 | -0.01 (-0.11, 0.09) | 0.88 |
|  | Sum score of 13 cytokines Age 14 months | 206 | -0.73 | 0.62 | -0.29 | -0.36 | -0.06 (-0.23, 0.11) | 0.48 |
|  | Ln AGP Age 28 months (g/L) | 164 | -0.9 | 0.34 | -0.32 | -0.29 | 0.03 (-0.06, 0.13) | 0.52 |
|  | Ln CRP Age 28 months (mg/L) | 333 | -0.64 | 0.69 | -0.29 | -0.34 | -0.05 (-0.28, 0.18) | 0.66 |
|  | Ln IFN-γ Age 28 months (pg/mL) | 322 | -0.66 | 0.68 | 1.73 | 1.7 | -0.03 (-0.12, 0.07) | 0.62 |
|  | Sum score of 13 cytokines Age 28 months | 322 | -0.66 | 0.68 | -0.3 | -0.55 | -0.25 (-0.58, 0.09) | 0.15 |
| N, 25th Percentile, and 75th Percentile are from the adjusted analyses  Adjusted for pre-specified and pre-screened covariates: the covariates mentioned in Supplementary Table 3.  Excludes children who had caregiver-reported recent illness in the past 7 days before sample collection. | | | | | | | | |
|  | | | | | | | | |

**Supplementary Table 12.** Enrollment Characteristics of Included and Excluded Mother-Child Dyads

|  |  |  | Included mother-child dyads (N=578)  n (%) or median (IQR) | Excluded mother-child dyads (N=324)  n (%) or median (IQR) |
| --- | --- | --- | --- | --- |
| Child |  | Female | 293 (51%) | 170 (52%) |
|  | Anthropometry (3 months) | Length-for-age Z score | -1.29 (-2.04, -0.48) | -1.31 (-2, -0.61) |
|  |  | -for-age Z score | -1.21 (-1.84, -0.51) | -1.23 (-1.98, -0.54) |
|  |  | Weight-for-length Z score | -0.26 (-1.19, 0.5) | -0.29 (-1.05, 0.39) |
|  |  | Head circumference-for-age Z Weight score | -1.83 (-2.49, -1.13) | -1.68 (-2.45, -0.89) |
|  | Anthropometry (14 months) | Length-for-age Z score | -1.44 (-2.22, -0.82) | -1.41 (-1.92, -0.72) |
|  |  | Weight-for-age Z score | -1.39 (-2.07, -0.8) | -1.21 (-1.77, -0.5) |
|  |  | Weight-for-length Z score | -1 (-1.68, -0.32) | -0.68 (-1.33, -0.1) |
|  |  | Head circumference-for-age Z score | -1.81 (-2.43, -1.23) | -1.75 (-2.37, -1.11) |
|  | Anthropometry (28 months) | Length-for-age Z score | -1.6 (-2.35, -1.01) | -1.41 (-2.06, -0.8) |
|  |  | Weight-for-age Z score | -1.57 (-2.13, -0.96) | -1.51 (-2.04, -0.83) |
|  |  | Weight-for-length Z score | -0.98 (-1.6, -0.36) | -1.06 (-1.59, -0.4) |
|  |  | Head circumference-for-age Z score | -1.79 (-2.37, -1.25) | -1.79 (-2.36, -1.22) |
|  | Diarrhea (14 months) | Caregiver-reported 7-day recall | 82 (16%) | 28 (11%) |
|  | Diarrhea (28 months) | Caregiver-reported 7-day recall | 42 (8%) | 17 (7%) |
|  | Acute respiratory illness (14 months) | Caregiver-reported 7-day recall | 154 (31%) | 73 (29%) |
|  | Acute respiratory illness (28 months) | Caregiver-reported 7-day recall | 131 (25%) | 52 (22%) |
| Mother |  | Age (years) | 24 (20, 27) | 23 (19, 26) |
|  |  | Gestational age (weeks) | 21.86 (17.29, 25.86) | 23.86 (19.71, 26) |
|  | Anthropometry at enrollment | Height (cm) | 149.93 (146.67, 153.71) | 151.1 (147.55, 153.95) |
|  | Education | Schooling completed (years) | 6 (4, 9) | 8 (5, 9) |
|  | Depression (14 months) | CESD-20^*^ score | 10 (6, 16) | 10 (6, 16) |
|  | Depression (28 months) | CESD-20^*^ score | 9 (5, 17) | 11 (6, 18) |
|  | Perceived stress (28 months) | Perceived Stress Scale score | 14 (11, 18) | 14 (10, 18) |
|  | Intimate partner violence | Any lifetime exposure | 288 (57%) | 117 (52%) |
| Household | Household Food Insecurity | Food-insecure households | 162 (28%) | 86 (27%) |

*CESD-20 = Center for Epidemiologic Studies Depression Scale Revised

**Supplementary Figures**

**
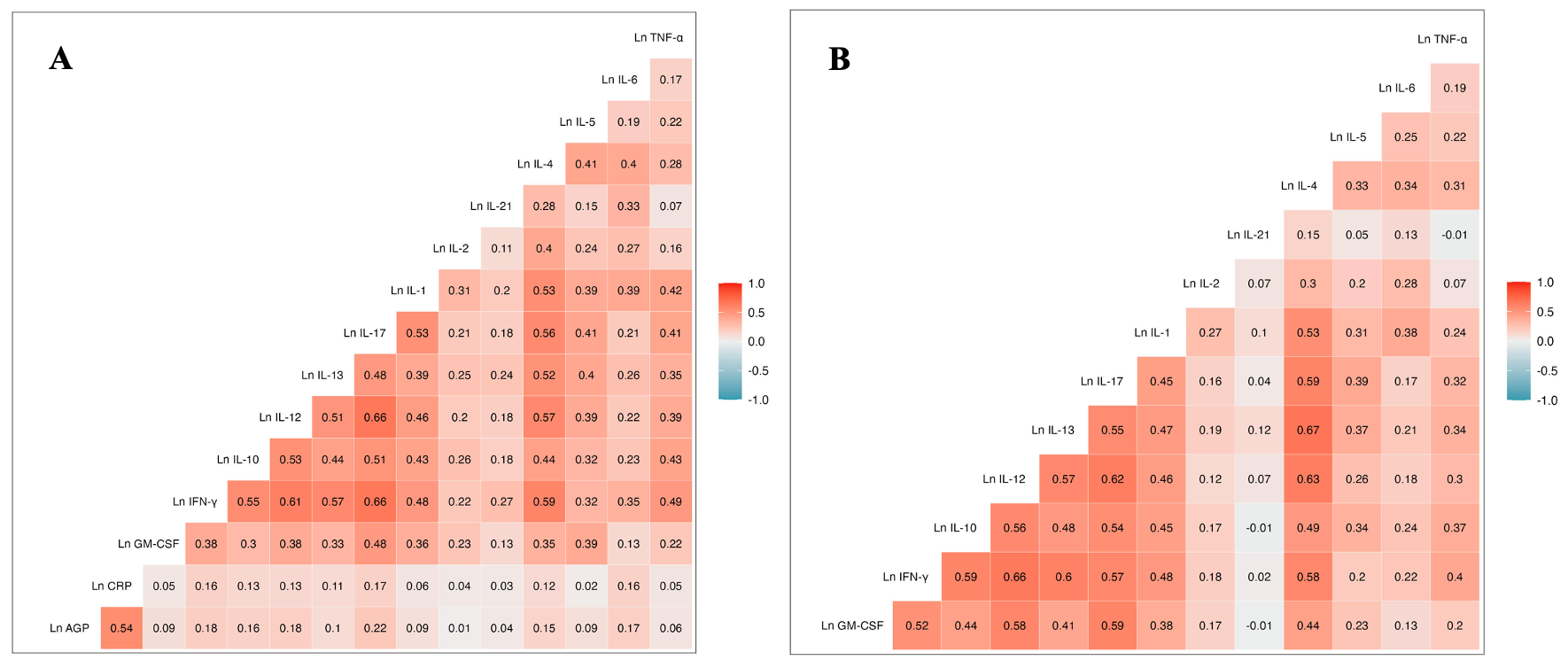
**

**Supplementary Figure 1.** Pearson Correlation Plots across the 13 cytokines included in the cytokine sum score from child cytokine measurements at Age 14 months (A) and 28 months (B).

**References**

1. Gelman A. Comment on “Post-hoc Power Using Observed Estimate of Effect Size is too Noisy to be Useful.” Annals of Surgery 2019;270:e64.

2. Larqué E, Morales E, Leis R, Blanco-Carnero JE. Maternal and Foetal Health Implications of Vitamin D Status during Pregnancy. ANM Karger Publishers; 2018;72:179–92.

3. Abbassi-Ghanavati M, Greer LG, Cunningham FG. Pregnancy and laboratory studies: a reference table for clinicians. Obstet Gynecol 2009;114:1326–31.

4. Kim HO, Kim H-S, Youn J-C, Shin E-C, Park S. Serum cytokine profiles in healthy young and elderly population assessed using multiplexed bead-based immunoassays. J Transl Med 2011;9:113.
